# Updating the Salivary Gland Transcriptome of *Phlebotomus papatasi* (Tunisian Strain): The Search for Sand Fly-Secreted Immunogenic Proteins for Humans

**DOI:** 10.1371/journal.pone.0047347

**Published:** 2012-11-06

**Authors:** Maha Abdeladhim, Ryan C. Jochim, Melika Ben Ahmed, Elyes Zhioua, Ifhem Chelbi, Saifedine Cherni, Hechmi Louzir, José M. C. Ribeiro, Jesus G. Valenzuela

**Affiliations:** 1 Laboratory of Transmission, Control and Immunobiology of Infectious Diseases, Pasteur Institute of Tunis, Tunis, Tunisia; 2 Vector Molecular Biology Section, Laboratory of Malaria and Vector Research, National Institute of Allergy and Infectious Diseases, National Institutes of Health, Rockville, Maryland, United States of America; 3 Faculty of Medicine of Tunis, Tunis El Manar University, Tunis, Tunisia; 4 Laboratory of Vector Ecology, Pasteur Institute of Tunis, Tunis, Tunisia; 5 Vector Biology Section, Laboratory of Malaria and Vector Research, National Institute of Allergy and Infectious Diseases, National Institutes of Health, Rockville, Maryland, United States of America; Centro de Pesquisas René Rachou, Brazil

## Abstract

**Introduction:**

Sand fly saliva plays an important role in both blood feeding and outcome of *Leishmania* infection. A cellular immune response against a *Phlebotomus papatasi* salivary protein was shown to protect rodents against *Leishmania major* infection. In humans, *P. papatasi* salivary proteins induce a systemic cellular immune response as well as a specific antisaliva humoral immune response, making these salivary proteins attractive targets as markers of exposure for this *Leishmania* vector. Surprisingly, the repertoire of salivary proteins reported for *P. papatasi*–a model sand fly for *Leishmania*-vector-host molecular interactions–is very limited compared with other sand fly species. We hypothesize that a more comprehensive study of the transcripts present in the salivary glands of *P. papatasi* will provide better knowledge of the repertoire of proteins of this important vector and will aid in selection of potential immunogenic proteins for humans and of those proteins that are highly conserved between different sand fly strains.

**Methods and Findings:**

A cDNA library from *P. papatasi* (Tunisian strain) salivary glands was constructed, and randomly selected transcripts were sequenced and analyzed. The most abundant transcripts encoding secreted proteins were identified and compared with previously reported sequences. Importantly, we identified salivary proteins not described before in this sand fly species.

**Conclusions:**

Comparative analysis between the salivary proteins of *P. papatasi* from Tunisia and Israel strains shows a high level of identity, suggesting these proteins as potential common targets for markers of vector exposure or inducers of cellular immune responses in humans for different geographic areas.

## Introduction

Phlebotomine sand flies are the vectors of *Leishmania* parasites, the causative agents of the tropical neglected disease leishmaniasis. During the blood-feeding process, sand flies inject saliva, which is composed of potent pharmacologically active components. These components, many of them proteinaceous, counteract the hemostatic and inflammatory system of the vertebrate host, allowing these insects to take a blood meal [1].The physiological changes induced by sand-fly saliva in host skin were shown to facilitate establishment of the *Leishmania* parasites in mice, ultimately producing a more severe disease manifestation [2], [3]. Conversely, development of an immune response to sand-fly salivary components or to bites of uninfected sand flies was shown to protect mice from *Leishmania* infection [3]–[5]. This protective immune response is proposed to be a T_H_1 cellular immune response specific to the salivary protein and is usually observed as a delayed-type hypersensitivity (DTH) response in the skin of animals [5]. Proteins that produce a T_H_2-biased response were not protective and, in some instances, exacerbated the disease outcome [5].

In humans living in endemic areas in Tunisia, peripheral blood mononuclear cells (PBMCs) isolated from individuals exposed naturally to *Phlebotomus papatasi* bites produced T_H_1-like and T_H_2-like responses after stimulation with *P. papatasi* salivary gland homogenate (SGH) [6]; however, the potentially protective salivary proteins have not been identified.

Furthermore, recent work conducted on a large cohort of individuals living in endemic areas of zoonotic cutaneous leishmaniasis showed that antibodies against *P. papatasi* saliva were highly prevalent in individuals naturally exposed to sand-fly bites [7]. These studies strongly suggest that sand-fly salivary proteins are potential targets to test human exposure to *P. papatasi* bites and to use them as epidemiologic tools to assess the risk of contracting this neglected disease. Previous work has identified potential immunogenic proteins from *P. papatasi* by western-blot analysis [7]; however, many of these immunogenic proteins were poorly represented in a Coomassie-stained SDS-PAGE [7]. Furthermore, it was shown in previous studies that proteomic or immunoproteomic analysis require the input of a parallel and complete transcriptomic analysis [8] to obtain desirable results, further supporting the need for a more comprehensive transcriptome analysis of this important vector.

*P. papatasi* is one of the most important vectors of *Leishmania major* in North Africa [9] and the Middle East and an important laboratory model to study sand fly-host-parasite interactions. The first attempt for a sand fly SG transcriptome to be described was that for *P. papatasi* [10]; it was limited to a small number of molecules and therefore lacking the necessary coverage to identify all or the majority of the salivary molecules from this vector. Later, extensive sequencing of eight other sand-fly species’ SG transcriptomes–including *Phlebotomus arabicus* [11], *Phlebotomus duboscqi* [12], *Phlebotomus ariasi* [13], *Phlebotomus perniciosus* and *Phlebotomus argentipes* [14]–established the abundance and diversity of molecules present in the Phlebotominae sialome. Findings from these transcriptomes suggest that there are still molecules in the saliva of *P. papatasi* that are yet to be identified. Because of the potential use of sand-fly salivary proteins as anti-*Leishmania* vaccines and as markers of sand-fly exposure in a *P. papatasi*-prevalent area, it is important to have a more comprehensive repertoire of the salivary molecules present in this sand-fly species. In the present study, the SG transcriptome of a colonized Tunisian strain of *P. papatasi* was sequenced and analyzed to further increase our knowledge of the sialome of this important vector of leishmaniasis.

## Results and Discussion

A cDNA library was constructed from the SGs of *P. papatasi* females (Tunisian strain) dissected 1 to 2 days post eclosion. From this cDNA library, 1900 random clones were selected and sequenced, resulting in 1603 high-quality sequences. These sequences were clustered together based on sequence homology and produced 99 contigs (with more than one sequence per contig) and 524 singletons (with only one sequence per contig). The presence of a signal peptide in the predicted proteins, indicative of extracellular secretion into the saliva, was analyzed using the SignalP server [15]. The majority of contigs assembled from three or more transcripts encoded a protein with a putative signal peptide sequence. It is important to note that the majority of contigs assembled from two or fewer transcripts were predicted to encode a cytoplasmic protein. This is probably due to the low coverage of the contig by this low number of sequences or to a 5′ truncated sequence that will appear not to have the signal secretory peptide. The most abundant transcripts were those coding for secreted proteins, suggesting most transcripts from this tissue target proteins for secretion. These abundant salivary transcripts were represented in 53 contigs with an average number of 5.48 sequences per contig and 41 singletons (total of 94 contigs coding for secreted proteins). All contigs and singletons were analyzed using the “basic local alignment search tool” (BLAST) to identify homology to other proteins in the non-redundant (NR) database, including the presence of conserved domains of the “simple modular architecture research tool” (SMART) [16], “protein families” (Pfam) [17] or SWISSP, “gene ontology” (GO), KOG, “conserved domain database” (CDD), or PRK databases.

We further selected and analyzed full-length transcripts coding for secreted proteins and grouped them by family (Table 1). We described the predicted molecular weight (mw), isoelectric point (pI), best match to the NR database, and the organism or sand fly with the highest homology. This allowed us to determine whether the molecules isolated in this cDNA library was described before in *P. papatasi* (Israeli strain) or represented a newly described molecule.

**Table 1:** Families of secreted proteins from salivary glands of *Phlebotomus papatasi* Tunisian strain.

					Putative mature protein				Best match to non-redundant database		
**Sequence name**	**Accession number**	**Contig** **number**	**Seq per** **contig**	**Transcript length**	**SigP**	**MW**	**pI**	**Protein** **Length (aa)**	**Best match**	**Species of** **best match**	**E-value**
**OBP SP12-like family of proteins**											
PPTSP12	JQ988874	Pp-38	24	545	Y	13.853	9.37	140	gi|15963505	*P. papatasi*	2E-075
PPTSP12	JQ988874	Pp-39	8	545	Y	13.853	9.37	140	gi|15963505	*P. papatasi*	2E-075
PPTSP12	JQ988874	Pp-40	5	534	Y	13.811	9.30	140	gi|15963505	*P. papatasi*	3E-073
PPTSP12	JQ988874	Pp-41	3	550	Y	13.827	9.39	140	gi|15963505	*P. papatasi*	9E-075
**OBP SP14.2-like family of proteins**											
*PPTSP14.2a*	JQ988876	Pp-90	7	517	Y	14.185	7.76	141	gi|112497698	*P. duboscqi*	7E-059
PPTSP14.2a	JQ988876	Pp-92	2	548	Y	14.110	7.13	141	gi|112497698	*P. duboscqi*	1E-052
*PPTSP14.2b*	JQ988877	Pp-97	5	534	Y	14.172	7.72	141	gi|112496839	*P. duboscqi*	2E-066
PPTSP14.2a	JQ988876	Pp-91	3	553	Y	14.159	6.48	141	gi|112497698	*P. duboscqi*	8E-060
**OBP SP14.5-like family of proteins**											
*PPTSP14.5*	JQ988878	Pp-30	13	536	Y	14.542	9.39	142	gi|112497496	*P. duboscqi*	2E-077
PPTSP14.5	JQ988878	Pp-29	7	534	Y	14.511	9.32	142	gi|112497496	*P. duboscqi*	2E-076
**OBP SP15-like family of proteins**											
PPTSP15	JQ988879	Pp-28	31	535	Y	14.502	9.39	142	gi|15963509	*P. papatasi*	2E-078
**OBP SP14-like family of proteins**											
PPTSP14	JQ988880	Pp-17	18	515	Y	14.736	8.85	142	gi|15963507	*P. papatasi*	8E-079
PPTSP14	JQ988880	Pp-16	11	731	Y	14.806	8.87	142	gi|15963507	*P. papatasi*	9E-080
PPTSP14	JQ988880	Pp-14	9	522	Y	14.764	8.86	142	gi|15963507	*P. papatasi*	1E-079
PPTSP14	JQ988880	Pp-22	9	513	Y	14.794	8.86	142	gi|15963507	*P. papatasi*	1E-078
PPTSP14	JQ988880	Pp-15	7	514	Y	14.736	8.85	142	gi|15963507	*P. papatasi*	8E-079
PPTSP14	JQ988880	Pp-13	3	514	Y	14.776	8.86	142	gi|15963507	*P. papatasi*	6E-079
PPTSP14	JQ988880	Pp-18	3	507	Y	14.722	8.85	142	gi|15963507	*P. papatasi*	6E-079
PPTSP14	JQ988880	Pp-19	3	514	Y	14.754	8.86	142	gi|15963507	*P. papatasi*	1E-078
PPTSP14	JQ988880	Pp20	3	553	Y	14.794	8.86	142	gi|15963507	*P. papatasi*	1E-078
PPTSP14	JQ988880	Pp-21	3	511	Y	14.720	8.85	142	gi|15963507	*P. papatasi*	3E-080
**OBP D7 SP28-like family of proteins**											
PPTSP28a	JQ988881	Pp-3	82	922	Y	27.365	9.04	254	gi|15963511	*P. papatasi*	1E-136
*PPTSP28b*	JQ988882	Pp-1	21	900	Y	27.258	8.96	254	gi|15963511	*P. papatasi*	1E-137
PPTSP28a	JQ988881	Pp-4	9	896	Y	27.304	8.70	254	gi|15963511	*P. papatasi*	1E-139
*PPTSP28c*	JQ988883	Pp-5	8	915	Y	27.315	8.43	254	gi|15963511	*P. papatasi*	1E-141
PPTSP28b	JQ988882	Pp-2	3	941	Y	27.229	9.09	254	gi|15963511	*P. papatasi*	1E-138
PPTSP28b	JQ988882	Pp-6	3	905	Y	27.309	8.57	254	gi|15963511	*P. papatasi*	1E-141
PPTSP28b	JQ988882	Pp-7	3	900	Y	27.336	8.43	254	gi|15963511	*P. papatasi*	1E-137
**OBP D7 SP30-like family of proteins**											
PPTSP30	JQ988884	Pp-101	1	870	Y	27.7	9.02	253	gi|15963513	*P. papatasi*	1E-146
**Yellow PPSP42-like family of proteins**											
PPTSP42	JQ988885	Pp-51	9	1330	Y	42.321	9.11	395	gi|15963517	*P. papatasi*	0.0
PPTSP42	JQ988885	Pp-52	3	1333	Y	42.385	9.07	395	gi|15963517	*P. papatasi*	0.0
**Yellow PPSP44-like family of proteins**											
PPTSP44	JQ988886	Pp-35	32	1335	Y	43.608	8.40	400	gi|15963519	*P. papatasi*	0.0
PPTSP44	JQ988886	Pp-34	17	1380	Y	43.667	8.58	400	gi|15963519	*P. papatasi*	0.0
**Antigen-5 PPSP29-like family of proteins**											
PPTSP29	JQ988887	Pp-64	9	1094	Y	28.844	9.10	272	gi|76589378	*P. papatasi*	1E-158
PPTSP29	JQ988887	Pp-67	7	1009	Y	28.673	9.04	272	gi|76589378	*P. papatasi*	1E-157
PPTSP29	JQ988887	Pp-68	6	1023	Y	28.93	9.04	272	gi|76589378	*P. papatasi*	1E-158
PPTSP29	JQ988887	Pp-66	3	1001	Y	28.884	9.16	272	gi|76589378	*P. papatasi*	1E-158
**Silk-related SP32-like family of proteins**											
PPTSP32	JQ988888	Pp-63	25	886	Y	24.465	8.95	246	gi|15963515	*P. papatasi*	1E-137
PPTSP32	JQ988888	Pp-62	11	883	Y	24.493	9.30	246	gi|15963515	*P. papatasi*	1E-134
PPTSP32	JQ988888	Pp-61	2	891	Y	24.519	8.95	246	gi|15963515	*P. papatasi*	1E-135
**SP34 protein. Family of sand-fly anticoagulant proteins**											
*PPTSP34*	JQ988889	Pp-73	8	1109	Y	34.07	9.21	313	gi|112496879	*P. duboscqi*	1E-143
**SP56.6-like family of proteins**											
*PPTSP56.6*	JQ988890	Pp-104	2	1537	Y	50.12	4.57	471	gi|299829444	*P. sergenti*	1E-172
**Alpha amylase family of proteins**											
*PPTAMY*	JQ988891	Pp-55	11	1727	Y	54.02	6.50	497	gi|4887104	*L.longipalpis*	0.0
**Apyrase SP36-like family of proteins**											
PPTSP36	JQ988892	Pp-76	8	1121	Y	35.90	9.03	336	gi|10443907	*P. papatasi*	0.0
PPTSP36	JQ988892	Pp-75	6	1105	Y	36.00	9.03	336	gi|10443907	*P. papatasi*	0.0
PPTSP36	JQ988892	Pp-77	6	1106	Y	35.91	9.03	336	gi|10443907	*P. papatasi*	0.0
**SP16-like family of proteins**											
*PPTSP14.3*	JQ988893	Pp-413	1	760	Y	14.06	4.82	159	gi|299829434	*P. sergenti*	8E-068
**SP2.5 kDa-like family of proteins**											
*PPTSP2.5*	JQ988875	Pp-147	1	1008	Y	3.1	10.6	49	gi|112497575	*P. duboscqi*	3E-050
**SP38.8 kDa-like family of proteins**											
*PPTSP38.8*		Pp-219	1	1022	Y	36.80	4.37	341	gi|299829376	*P. tobbi*	2E-050

(Only full-length sequences are shown in this table. Transcripts not described before are in italics).

The primary objective of this work was identification of secreted proteins from the SG of *P. papatasi* that can potentially be used as vaccine candidates or as markers of vector exposure in sand fly-prevalent areas. Additionally, by determining the degree of homology between molecules from Israeli and Tunisian strains of *P. papatasi,* we could identify redundant vaccine or peptide markers across different locations. Finally, we wished to gain a deeper understanding of the repertoire of proteins present in the SGs of *P. papatasi*.

The following are the most abundant and representative families of secreted proteins we identified in this work, including several salivary proteins not described before in *P. papatasi*.

### OBP/D7 Superfamily–OBP Family D7-Related Proteins

D7 was an arbitrary name given to one of the first salivary proteins cloned from the mosquito *Aedes aegypti* [18]. Later, homologs of the D7 protein were identified in the saliva of anopheline mosquitoes, sand flies, black flies [19], and biting midges [20]. The D7 protein was later found to belong to the superfamily of pheromone/odorant binding proteins (OBP) [21]. Only recently was the function of mosquito salivary D7 proteins elucidated [22], [23]. Some of the mosquito D7 proteins were shown to bind biogenic amines and others to work as anticoagulants [23], [24].

The most abundant sequences in this cDNA library encode for members of the D7 proteins. We identified transcripts coding for proteins sharing 91% identity with the secreted D7 protein of 28 kDa (AAL11048) from *P. papatasi* Israeli strain. The identified protein (PPTSP28) corresponds to the most abundant transcript found in the current *P. papatasi* SG cDNA library as represented by the number of contigs and the number of transcripts per contig (Table 1). The mature protein (PPTSP28) has a predicted mw of 27.3 kDa and a predicted pI of 9.0. It is important to note that we show only the contigs containing three or more transcripts due to the fact that some of the contigs with two sequences or fewer contained 5′ truncated transcripts. The transcripts present in the different contigs of PPTSP28 on Table 1 represent probable alleles of this gene. This could explain the slightly different mw and pI for the PPTSP28 molecules shown by the different contigs.

We found one contig (Pp-101) with one transcript coding for a D7-related protein of 30 kDa. PPTSP30 shares 98% identity with the D7 (30 kDa) from *P. papatasi* Israeli strain (AAL11049). PPTSP30 has a predicted mw of 27.7 kDa and a basic pI of 9.02 (Table 1).

**Figure 1:**
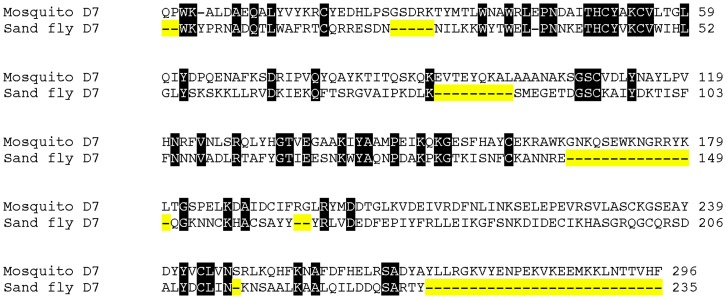
Alignment of *Anopheles stephensi* D7 protein (ANST D7L1) and the sand fly D7 protein (PPTSP28a) from *Phlebotomus papatasi* Tunisian strain. Black background shading represents identical amino acids. Yellow background shading represents amino acids absent in the sand fly D7 compared with the An. stephensi D7 protein.

In mosquitoes, there are two types of D7 proteins, the D7 long form (34–37 kDa) and the D7 short form (15–20 kDa) [25]. The D7 protein from sand flies resembles the mosquito long form; however, there is only 26% amino acid (aa) identity between these two proteins (Figure 1). Furthermore, the D7 protein from sand flies is missing a portion of the protein in the middle and at the carboxy terminal region (Figure 1). This makes the sand-fly salivary proteins a bit smaller than mosquito D7 long-form proteins with a maximum mw between 27.3 to 27.7 kDa. Therefore, we propose that this family of proteins in sand flies be referred as a medium form of D7 proteins.

**Figure 2:**
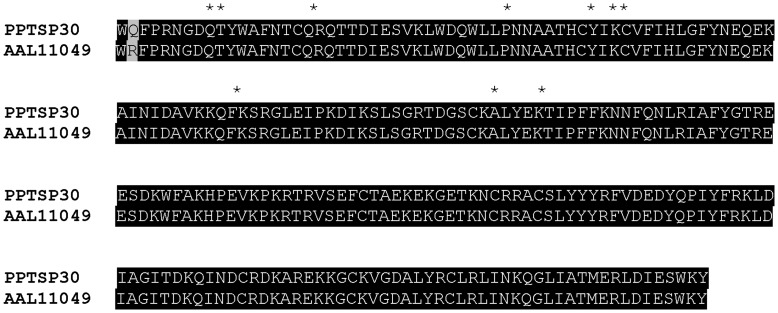
Alignment of PPTSP30 from *Phlebotomus papatasi* Tunisian strain and the D7 protein from *P. papatasi* Israeli strain (AAL11049). Black background shading represents identical amino acids. *Indicates the essential amino acids for leukotriene binding activity in mosquito D7 protein.

It was recently shown that the *Anopheles stephensi* mosquito D7 protein (long form) AnSt-D7L1 binds thromboxane A2 and cysteinyl leukotrienes [22]. The essential amino acids responsible for this binding in the mosquito D7 [22] are also present in the D7 protein from sand flies (Figure 2, asterisks) suggesting that sand-fly D7 proteins (both PPTSP28 and PPTSP30) may bind thromboxane A2 and cysteinyl leukotrienes.

**Figure 3:**
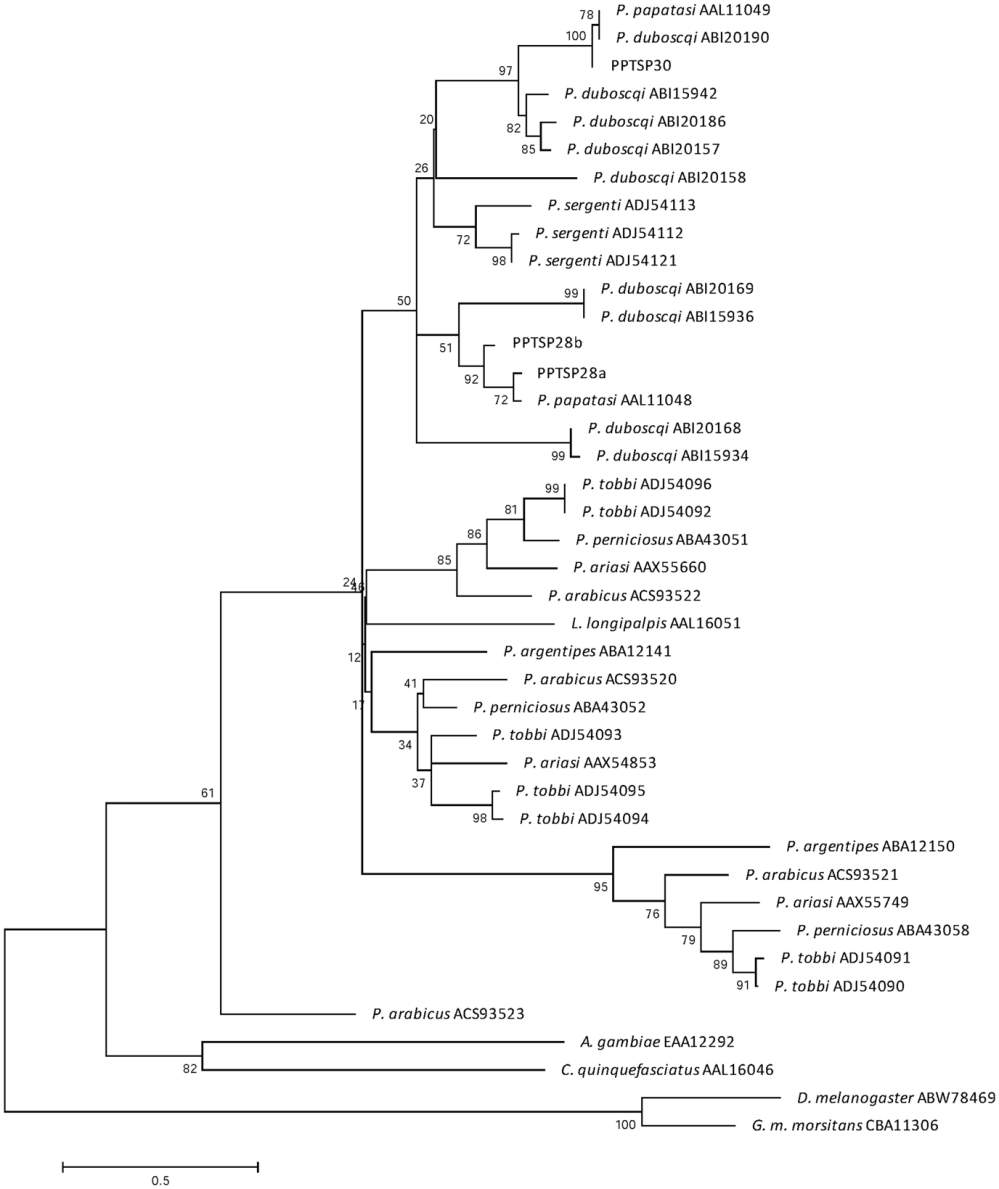
Phylogenetic analysis of sand fly salivary proteins of two D7 salivary proteins identified in this cDNA library (PPTSP28 and PPTSP30) and the D7 salivary proteins from *Phlebotomus papatasi* Israeli strain, *P. duboscqi, P. sergenti, P. tobbi, P. perniciosus, P. ariasi, P. arabicus, Lutzomyia longipalpis*, and *P. argentipes*, and other Diptera including *Drosophila melanogaster, Glossina morsitans morsitans, Culex quinquefasciatus*, and *Anopheles gambiae*. Accession numbers are next to each species, and node values indicate branch support.

Phylogenetic tree analysis of the sand-fly D7 proteins, and the D7 from other insects, shows that D7 from sand flies are clustered apart from mosquito, *Drosophila*, and *Glossina* D7 proteins (Figure 3). Furthermore, PPTSP28 and PPTSP30 are clustered into a large clade that includes D7 from sand flies of the subgenus *Phlebotomus* and *Paraphlebotomus*, such as *P. papatasi*, *P. duboscqi,* and *P. sergenti*, and apart from sand flies from the subgenus *Larroussius*, *Euphlebotomus*, and *Adlerius*. PPTSP30 is grouped together with 30-kDa D7 of from *P. papatasi* and *P. duboscqi* (Figure 3). This analysis also suggests that three different related D7 proteins found in this transcriptome–PPTSP28a, PPTSP28b, and PPTSP28c–are probably a case of gene duplication. PPTSP28a and PPTSP28b are clustered together with the D7 protein of 28 kDa from *P. papatasi* Israeli strain and *P. duboscqi*. PPTSP28b is more closely related to the D7 protein from *P. papatasi* Israeli strain than it is to PPTSP28a (Tunisian strain).

### Small Members of the OBP Family–SP12-, SP14-, and SP15-like

**Figure 4:**
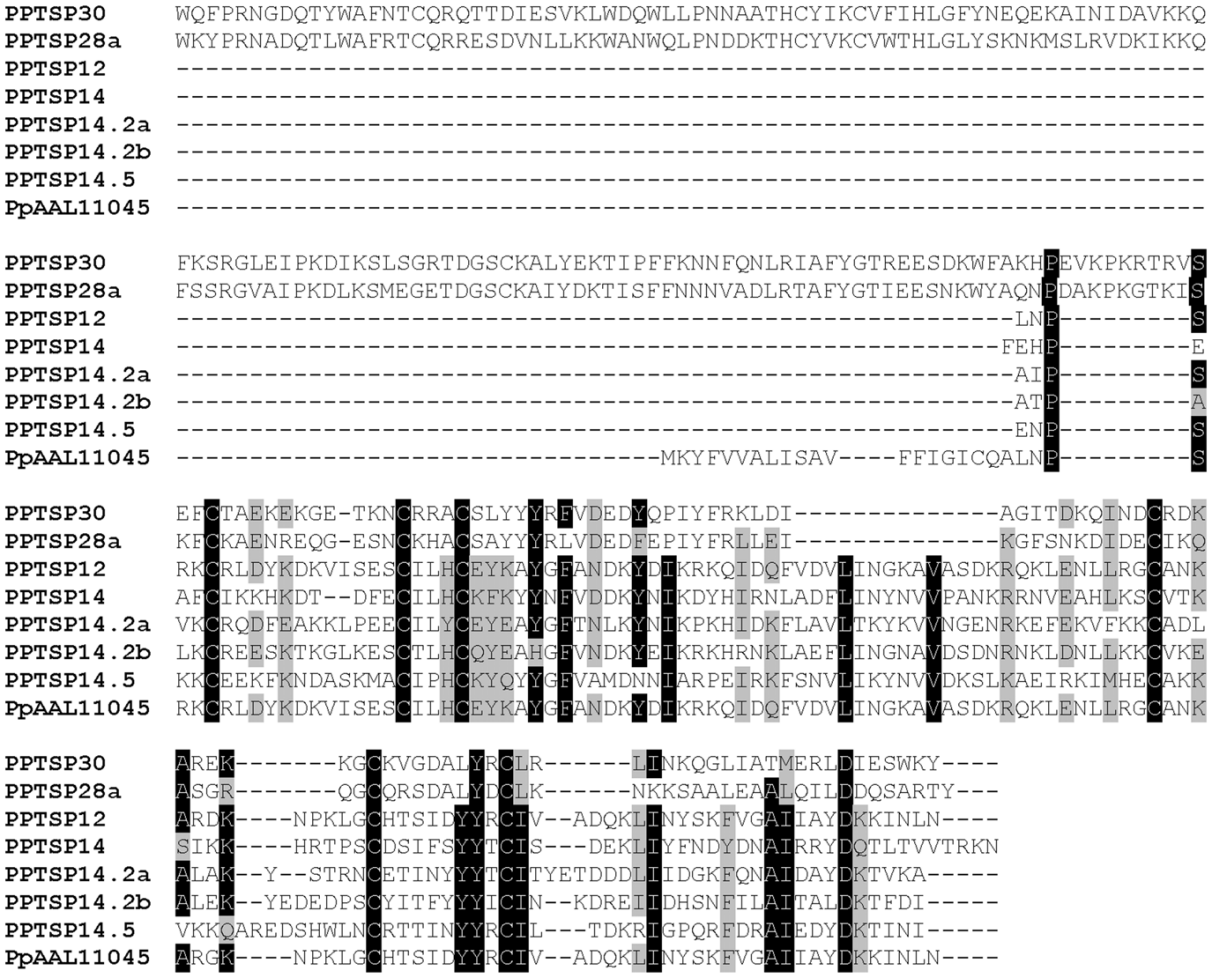
Multiple sequence alignment of the large OBP (D7 related proteins PPTSP30 and PPTSP28) and the small molecular weight OBP from the salivary glands of *Phlebotomus papatasi*.

One of the first molecules to be characterized from *P. papatasi* was the salivary protein SP15 [10]. This protein was shown to be immunogenic and conferred protection in mice against *L. major* infection [5], [10]. The biologic function of this protein remains unknown [14]. Furthermore, other proteins with similar sequence but different molecular weight, including SP12 and SP14 proteins, were also identified in this and other sand-fly species [14]. Later, it was found that these proteins are members of OBP, similar to the D7 family with smaller molecular weight. This family of proteins appears to derive from an ancestral OBP, which has since evolved to the short form of D7 salivary proteins in mosquitoes and the D7 protein in sand flies [25]. The protein sequence of this family of proteins between different insects is very divergent, and generally only cysteines are highly conserved [20]. In *P. papatasi,* there are three reported families of these types of proteins, the 12-kDa (PpSp12), the 14-kDa (PpSP14), and the 15-kDa (PpSp15). Historically, the name was given according to the molecular weight first observed for these proteins on an SDS-PAGE from the SGs of *P. papatasi* [10]; however, their molecular weight (predicted by their transcripts) may vary depending on the sand-fly species [14]. Therefore, we will refer to them as SP12-like, SP14-like, and SP15-like families of proteins. Sequence alignment between members of this family of small molecular weight proteins and the sand-fly D7 family of proteins (PPTSP28 and PPTSP30) shows that these proteins are related, and the small OBP proteins align from the middle of the D7 protein toward the carboxy terminal region (Figure 4). Few amino acids are conserved, and cysteines are highly conserved. The small OBPs from sand flies may have resulted from a gene duplication event of the medium D7 protein where the N terminal region was lost in the process. This also supports the hypothesis that sand-fly D7 and the small molecular weight proteins (SP12-, SP14-, and SP15-like) belong to the family of OBPs. The function of these small molecular OBPs remains to be elucidated.

#### OBP PpSP12-like

Transcripts coding for the 12-kDa protein (PPTSP12) are very abundant in this cDNA library (Table 1). PPTSP12 has two potential N-glycosylation sites at positions 22 and 122 as predicted by the NetNGlyc server. These proteins share 99% identity with the 12-kDa salivary protein of *P. papatasi* Israeli strain (gi|15963505) and 82% identity with the 13.7-kDa salivary protein from *P. duboscqi* (gi|112497317). The predicted mw of PPTSP12 is 13.8 kDa with a pI of 9.4.

#### OBP PpSP14-like

Transcripts coding for a 14-kDa secreted protein were the most abundant from this family of small OBP proteins (Table 1). The PPTSP14 protein shares 97% identity with the previously reported 14-kDa of *P. papatasi* Israeli strain (gi|15963507). The estimated mw of PPTSP14 is 14.7 kDa with a basic pI of 8.8.

#### OBP PpSP15-like

This group of proteins is similar to the PpSP15-like family of proteins present only in the saliva of sand flies, suggesting that this family was a specific invention that occurred during sand-fly evolution [14]. Thus far, the SP15 family of proteins has been reported as the most abundant protein family in most sand-fly species, although in this cDNA library, this transcript is not the most abundant; PPTSP14 transcripts are at least 2 fold more represented than PPTSP15 (Table 1). PPTSP15 was represented by 4 contigs with an average number of 8.5 sequences per contig. This protein shares 99% identity with the previously described SP15 protein of *P. papatasi* Israeli strain (gi|15963509).

#### OBP-SP14.5 kDa-like

This is the first description of this member of the OBP family of proteins in *P. papatasi*. The 14.5 kDa-like proteins were first identified in *P. duboscqi* [12]. PPTSP14.5 is represented by two contigs (average number of ten sequences per contig) and shares 97% aa identity with the 14.5-kDa salivary protein of *P. duboscqi.*

#### OBP-SP14.2 kDa-like

**Figure 5:**
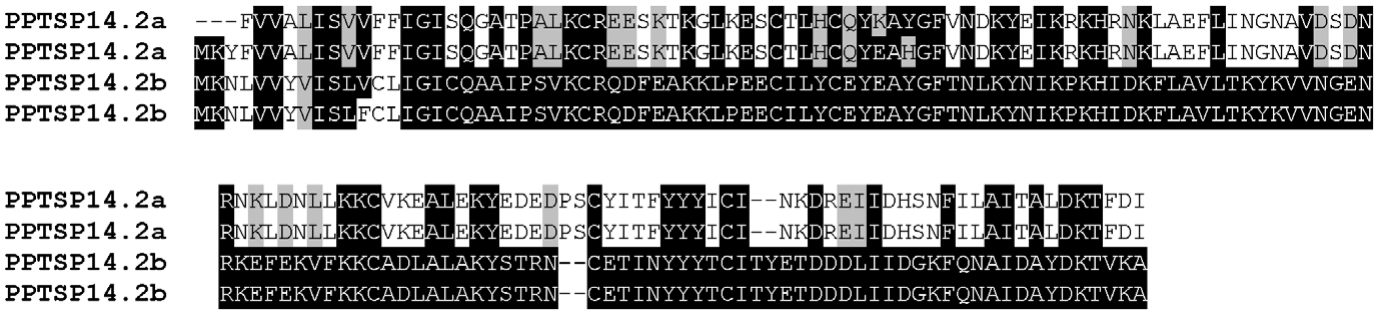
Multiple sequence alignment of the two distinct members of the PPTSP14.2 family of proteins from the saliva of *Phlebotomus papatasi*. Black shading represents identical amino acids.

The 14.2-kDa family of proteins was first described in *P. duboscqi* and not previously identified in *P. papatasi* SGs [12]. PPTSP14.2 proteins were represented by nine contigs with an average number of 2.66 sequences per contig. PPTSP14.2 is similar to the 14.2-kDa salivary protein of *P. duboscqi* (gi|112496839). We found two distinct members of this family in this cDNA library: PPTSP14.2a and PPTSP14.2b. These two proteins have significant differences at the amino acid level (Figure 5). PPTSP14.2a is more related to the 14.2-kDa from *P. duboscqi* Mali strain, and PPTSP14.2b is more related to the 14.2-kDa from *P. duboscqi* Kenya strain (data not shown).

**Figure 6:**
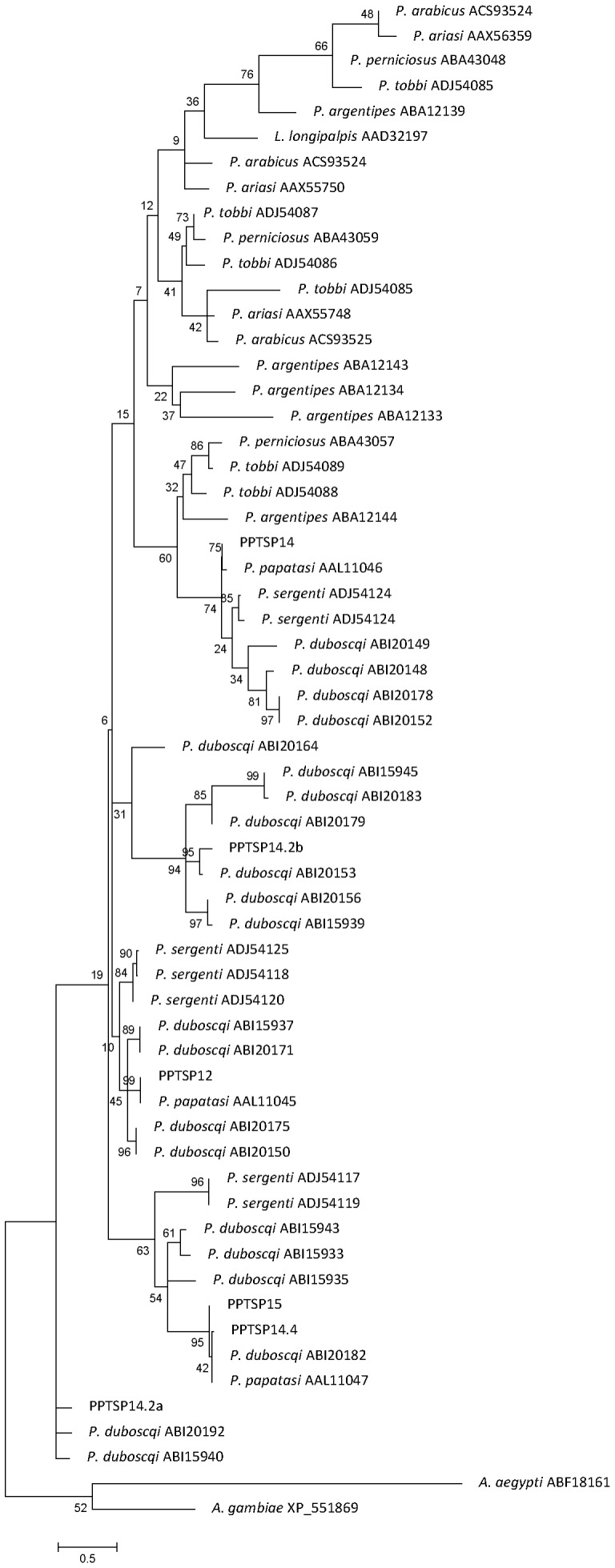
Phylogenetic analysis of the small OBP family of proteins from *Phlebotomus papatasi* Israeli strain (Pp), *P. duboscqi* (Pd), *P. sergenti* (Ps), *P. tobbi* (Pt), *P. perniciosus* (Pp), *P. ariasi* (Pa), *P. arabicus* (Pa), *P. argentipes* (Pa), and *Lutzomyia longipalpis* (Ll), *Aedes aegypti* (Aa), and *Anopheles gambiae* (Ag). Accession numbers are next to each species designation, and node values indicate branch support.

Based on this newly gathered information, we can suggest that *P. papatasi* has five members of the small OBP family of protein: SP12-, SP14-, SP14.2-, SP14.5-, and SP15-like. Phylogenetic tree analysis of these proteins show these molecules are clearly separated in different clades (Figure 6), and they are more closely associated to *P. papatasi* and *P. duboscqi* small OBP proteins than OBP from other sand-fly species.

### Antigen 5 Family of Proteins

Antigen 5 belongs to the CAP (CRISP, Ag5, PR-1) family of proteins present in the saliva of most blood-sucking insects and also in hookworms. The function of this family of proteins remains to be elucidated. We identified eight contigs with transcripts coding for proteins related to a *P*. *papatasi* 29-kDa secreted salivary protein (gi|76589378) that belongs to the Antigen 5-related proteins (Table 1). PPTSP29 has 1 potential N-glycosylation site and 27 potential O-glycosylation sites. PPTSP29 showed 96% identity with the previously described 29-kDa protein of *P. papatasi* Israeli strain (gi|76589378) and only 63% identity with the antigen 5-related protein from *Lutzomyia longipalpis* (gi|4887102). PPTSP29 has a predicted mw of 28.8 kDa with a pI of 9.1.

### SP32 kDa-Like Proteins

PpSP32-like family of proteins, previously identified in *P. papatasi* SGs, is similar to a silk protein from *Nephila clavipes* [10]. Of all blood-feeding insects studied to date, this family of proteins was only found in the saliva of sand flies [11]. The function of this protein remains unknown. We found three contigs coding for PPTSP32 in this library. PPTSP32 showed significant identity with the 32-kDa salivary protein from *P. papatasi* Israeli strain (gi|15963515). PPTSP32’s predicted mw is 24.4 kDa with a pI of 8.9. Forty-seven O-glycosylation sites were predicted in this molecule, and no N-glycosylation sites were present, suggesting this protein may be a mucin. The discrepancy between the previously reported molecular weight of 32 kDa that was observed by SDS-PAGE [10] and the one predicted by the transcript (24.4 kDa) is probably due to the post translational modification (O-glycosylations) identified in this molecule.

### Sp34-kDa Lufaxin Family of Sand-Fly Salivary Anticoagulant

**Figure 7:**
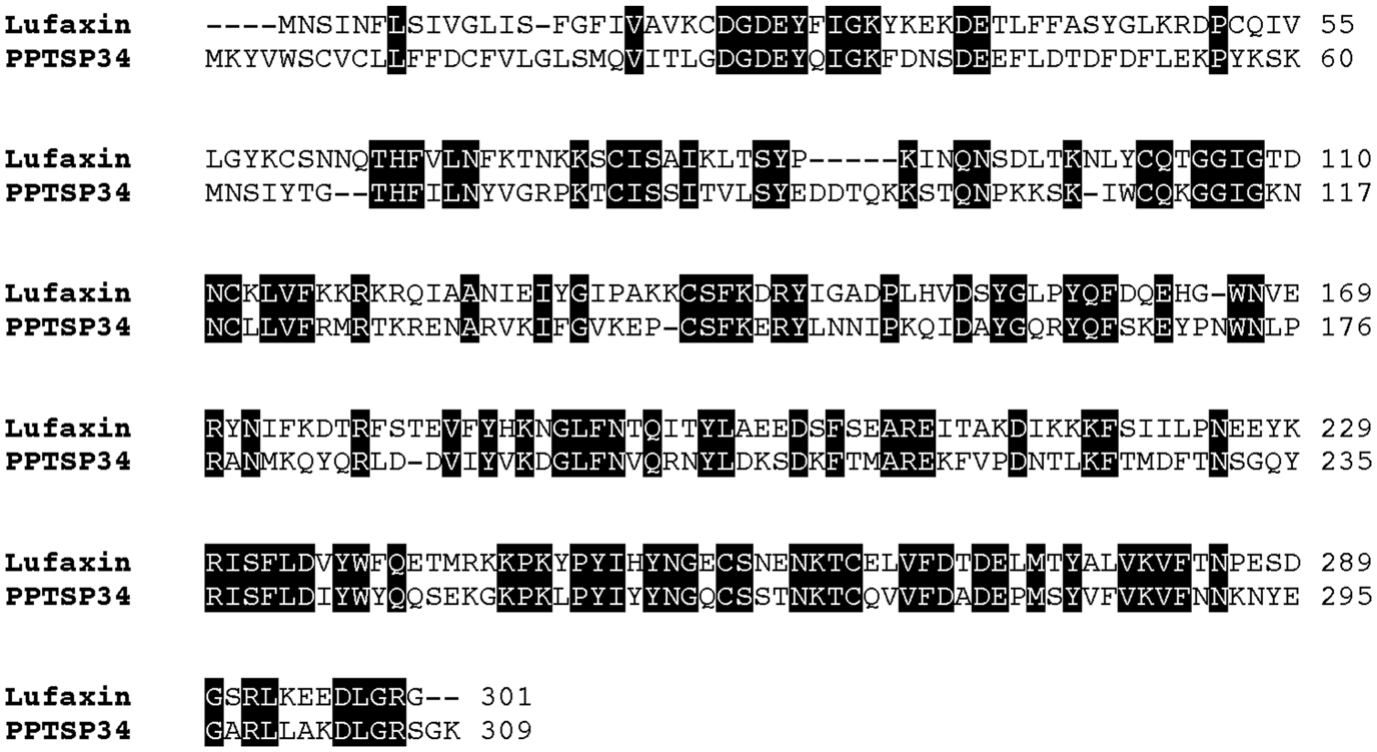
Alignment of Lufaxin, the Factor Xa inhibitor from the saliva of *Lutzomyia longipalpis*, and the PPTSP34 salivary protein from *Phlebotomus papatasi* Tunisian strain. Black background shading represents identical amino acids.

Lufaxin is a salivary protein from the sand fly *Lu. longipalpis* that was recently demonstrated to be a specific inhibitor of Factor Xa and of the activation of PAR2 [26]. The sequence of Lufaxin has been reported in other sand-fly species but not in *P. papatasi* [26]. This is the first report of this family of proteins in *P. papatasi* SGs. PPTSP34 shares a good level of identity to Lufaxin (Figure 7), suggesting PPTSP34 is also an inhibitor of Factor Xa. PPTSP34 appears to be sand-fly specific, consistent with previous findings [11]. Apart from four sand-fly species, we did not find any significant matches of PPTSP34 with any other proteins in accessible databases, suggesting this family of anticoagulants is only present in sand flies.

### Apyrase

Apyrases are enzymes that hydrolyze ADP, a key agonist for platelet aggregation. Sand-fly apyrases belong to the *Cimex* apyrase family of proteins and are distinct from the apyrases in mosquitoes, which belong to the 5′ nucleotidase family of proteins [27]. The predicted mw of *P. papatasi* apyrase (PPTSP36) is 36 kDa and the predicted pI is 9.03.

Sequence alignment of *P*. *papatasi* apyrase (PPTSP36) shows 98% identity with the salivary apyrase from *P*. *papatasi* Israeli strain (gi|10443907).

### Yellow-Related Proteins (PPTSP42 and PPTSP44)

The gene coding for a yellow protein was first described in *Drosophila melanogaster* [28]. The proteins in this family appear to derive from a common ancestor of the major royal jelly proteins from honeybees and the yellow protein from *Drosophila spp*.

Two clusters contained transcripts coding for a secreted yellow protein of 42 kDa. This protein has 98% aa identity with the 42-kDa salivary protein from *P. papatasi* Israeli strain (gi|15963517). The predicted mw of PPTSP42 is 42 kDa with a predicted basic pI of 9.1.

**Figure 8:**
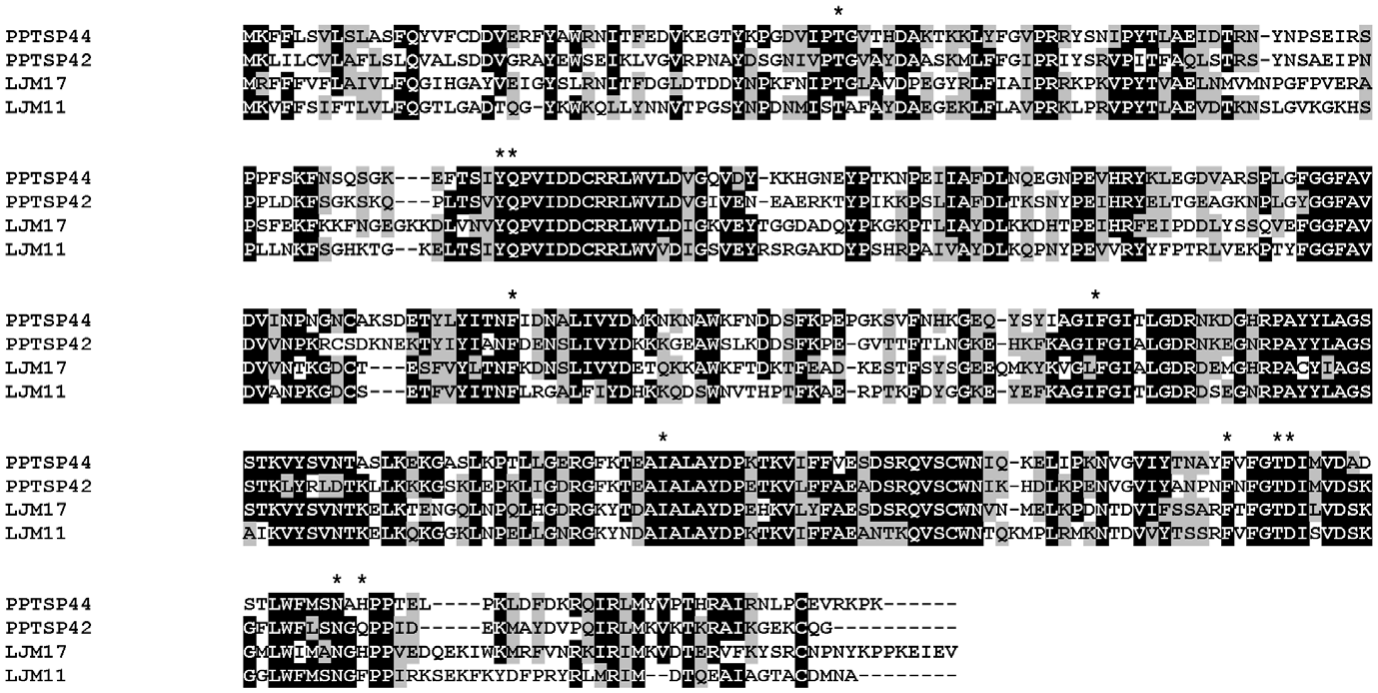
Multiple sequence alignment of yellow-related proteins from *Phlebotomus papatasi* PPTSP42 and PPTSP44 and two yellow proteins from *Lutzomyia longipalpis* LJM11 and LJM17. Black shading represents identity. *Indicates essential amino acids for LJM11 and LJM17 binding of biogenic amines.

We also identified a second member of this protein family, a yellow protein of 44 kDa that shares 98% identity to the 44-kDa yellow protein from *P. papatasi* Israeli strain (gi|15963519). The sand-fly yellow-related proteins are also similar to the yellow-B protein from *D. melanogaster* with unknown function and to the major royal jelly protein from *Apis mellifera* [29]. Apparently, sand flies are the only blood-sucking insect that has a yellow-related protein in their SGs [30]. The function of this family of proteins was first described from a yellow protein from the sand fly *Lu. longipalpis* [30]. The yellow-related proteins from *Lu. longipalpis*–LJM11, LJM17, and LJM111–function as biogenic amine-binding proteins. Although the identity between the yellow proteins from *Lu. longipalpis* (LJM11 and LJM17) and the two yellow proteins identified in this cDNA library (PPTSP42 and PPTSP44) is not very high (Figure 8), the amino acids responsible for the binding to serotonin are highly conserved, suggesting that the two yellow proteins from *P. papatasi* may also have biogenic amine-binding function. Another activity was recently reported from LJM111 [31]. This protein functions as an anti-inflammatory and anti-arthritis molecule by acting directly in dendritic cells [31]. Further tests are needed to determine whether *P. papatasi* yellow proteins also have this anti-inflammatory activity.

### SP56.6-Like Family of Proteins

One transcript coding for a putatively secreted 50-kDa salivary protein (gi|299829444) belonging to the 56.6-kDa protein family previously described in *P. sergenti* was present in this cDNA library (Table 1). This protein has not been reported in *P. papatasi* saliva. The mature protein has a predicted mw of 50.12 kDa with a pI of 4.57.

### SP38.8/*Aegyptin*-Like Family of Proteins

One transcript coding for a putative secreted protein of 39 kDa and belonging to the SP38.8-like protein (gi|299829376) previously reported in *Phlebotomus tobbi* was identified in this cDNA library (Table 1). This protein is similar to the Aegyptin family of proteins reported in *Ae. aegypti* [32]. Aegyptin was shown to block collagen-induced human platelet aggregation by binding to collagen [32], suggesting that PPSP38.8 may also inhibit collagen-induced platelet aggregation. This protein has not been previously reported in *P. papatasi* saliva. The mature protein has a predicted mw of 39.8 kDa with a pI of 4.45.

### SP16 Family of Proteins

**Figure 9:**
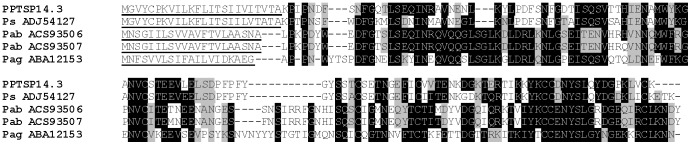
Multiple sequence alignment of the putative PPTSP14.3 protein and the SP16-like salivary proteins from *Phlebotomus sergenti* (Ps), *Phlebotomus arabicus* (Pab), and *Phlebotomus argentipes* (Pag). Underlining indicates the predicted signal peptide sequence; black shading represents identity between amino acids of the predicted mature peptide.

One transcript coding for a putatively secreted 16-kDa salivary protein (gi|299829434) belonging to the 14.3-kDa protein family was present in this cDNA library (Table 1). This protein has not been reported in *P. papatasi* saliva. PPTSP16 has 2 potential N-glycosylation sites and 25 O-glycosylation sites. PPTSP16 shows 39% identity with the previously described 14.3-kDa protein from *P. sergenti* (gi|299829434), 38% identity with the 16-kDa salivary protein SP73 of *P. argentipes* (gi|74486577), 35% with 16-kDa salivary protein A (gi|242564737), and 34% with 16-kDa salivary protein B (gi|242564754) from *P. arabicus* (Figure 9). The mature protein has a predicted mw of 14 kDa with a pI of 4.8.

### 2.5-kDa Family of Proteins

**Figure 10:**
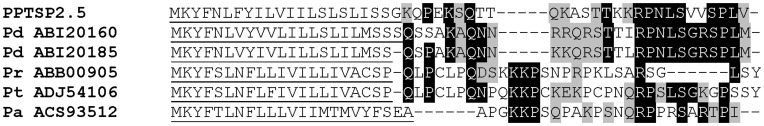
Multiple sequence alignment of PPTSP2.5 and the SP2.5-like proteins from *Phlebotomus duboscqi* (Pd), *Phlebotomus perniciosus* (Pr), *Phlebotomus tobbi* (Pt) and *Phlebotomus arabicus* (Pa). Underlining indicates the predicted signal peptide sequence; black shading represents identity between amino acids of the predicted mature peptide.

This family of proteins is the smallest reported so far from the saliva of sand flies. This is the first account for this family of proteins in *P. papatasi*. We identified one transcript coding for this family of proteins (Table 1). PPTSP2.5 has 4 potential N-glycosylation sites and 12 potential sites for O-glycosylation. PPTSP2.5 is 56% and 50% identical to the 2.5-kDa salivary protein (gi|112497575) and the 2.8-kDa salivary protein (gi|112496993) from *P. duboscqi,* respectively. Multiple sequence alignment (Figure 10) shows that this protein has some similarity but no significant homology with the 2.7-kDa protein from *P. perniciosus* (gi|77696453), the 3.7-kDa protein from *P. tobbi* (gi|299829392), and the 2.7-kDa from *P. arabicus* (gi|242564868). Our finding confirms the suggestion that the 2.7-kDa peptides are nonspecific for the subgenus *Larroussius* [14]. The predicted mw of the mature protein is 3.09 kDa with a predicted basic pI of 10.58. The function of this protein remains unknown.

### 5′-Truncated Sequences of Potentially Secreted Proteins

We found a number of low-abundance 5′-truncated transcripts coding for potentially secreted proteins previously described as secreted proteins in other sand-fly species–the salivary pyrophosphatase previously reported in *P. argentipes* (gi|74486581) and the salivary endonuclease (gi|76446619) reported in *P. perniciosus.*

### Proteins with Other Activities Not Involved in Blood Feeding

In the current cDNA library, four clusters coding for putatively secreted alpha amylase were found. This putative alpha amylase was found to be highly homologous to the amylase from *Lu. longipalpis* (gi|4887104) [33], *Scaptodrosophila lebanonensis* (gi|21954516), and *Drosophila kikkawai* (gi|7768505). Alpha-amylase activity was previously described in homogenates of young, unfed, male and female *P. papatasi* and in the gut and salivary preparations of these sand flies [34]. This enzyme hydrolyzed dietary starch, the major component in the natural diet of *P. papatasi*, to maltose, which is then cleaved to glucose by alpha glucosidase [34]. The identified amylase in this cDNA library may account for these reported activities.

There were numerous transcripts coding for potentially secreted proteins with high homology to other molecules previously described in the gut of sand flies (Table 2). These include a peritrophin-like protein from *P. papatasi* (gi|15736159), a 11.6-kDa protein, a 13.6-kDa protein previously reported in the midgut transcriptome of *P. papatasi* [35], PpGalectin–shown to be the receptor for parasites in the midgut of *P. papatasi* [36], two microvillar-like proteins, and a midgut trypsin, among others [35]. These transcripts, previously shown to be present in the midgut of other sand flies, probably derive from contaminant tissue during the dissection of SGs and most likely do not represent transcripts from SGs. Contrariwise, all of the SG associate molecules identified in this transcriptome have never been identified in sand fly midgut transcriptomic analyses [35]. TIMP-3-like proteins were previously identified in the SG transcriptome of *Glossina morsitans* [37] and whole transcriptomes of *Ae. aegypti* (gi|157136338) and *Culex quinquefasciatus* (gi|170034292). This is the first account for this family of tissue inhibitor of metalloproteases proteins (TIMP) in sand flies. The predicted mw of PPTTIMP3 protein is 20.65 kDa with a predicted basic pI of 9.33. There are three potential N-glycosylation sites and 28 potential O-glycosylation sites. This protein can be working as an angiogenic inhibitor or an immunosuppressant due to the Netrin domain present in this protein. Because of the lack of presence of this type of protein in the saliva of other sand flies, and the presence of midgut transcript in this cDNA library, it may also be possible that this type of protein does not derive from the SGs but from the midgut or another organ. Further tests are needed to confirm the specific expression of this transcript in SG of this sand fly.

**Table 2:** Non-salivary gland proteins. Potentially midgut or other organs proteins.

						Best match to non-redundant database		
Sequence name	Contig Number	Seq per contig	SigP	mw	pI	Best Match	Species of best match	E-value
**Midgut proteins**								
PPT11.6	Pp-202	1	N	11.9	9.27	gi|157361609	*P. papatasi*	3.00E-53
PPT13.6	Pp-566	1	Y	11.598	10	gi|157361609	*P. papatasi*	2.00E-65
PPTmicrovilli-like	Pp-404	1	Y	23.707	5.28	gi|157361605	*P. papatasi*	1.00E-118
PPTmicrovilli-like	Pp-565	1	Y	23.779	5.43	gi|157361605	*P. papatasi*	1.00E-119
**Extracellular matrix**								
PPTGalectin	Pp-493	1	N	10.491	8.66	gi|47121805	*P. papatasi*	0.016
**Serine protease**								
PPTtrypsin 1	Pp-115	1	Y	28.442	5.03	gi|32394738	*P. papatasi*	1.00E-148
**Peritrophin-like protein**								
PPTperitrophin-like	Pp-102	1	Y	9.648	4.4	gi|157361591	*P. papatasi*	8.00E-47
**Hypothetical protein**								
PPTAND_04019	Pp-614	1	N			gi|312383031	*An. darlingi*	2.00E-11
PPTAaeL008425	Pp-478	1	N	15.322	5.29	gi|157118844	*Ae. aegypti*	1.00E-65
PPTAaeL012123	Pp-376	1	Y	18.615	9.92	gi|157131504	*Ae. aegypti*	2.00E-95
PPTAND_22328	Pp-223	1	N			gi|312371260	*An. darlingi*	5.00E-27
**Conserved hypothetical protein**								
PPTH1	Pp-524	1	N			gi|170069526	*C. quinquefasciatus*	1.00E-43
PPTH2	Pp-195	1	N			gi|170032716	*C. quinquefasciatus*	3.00E-40
**Other proteins**								
PPTAGAP012418-PA	Pp-426	1	N			gi|58393517	*An.gambiae*	1.00E-14
PPTFAM8A1	Pp-608	1	N			gi|170036645	*C. quinquefasciatus*	6.00E-51
PPT25	Pp-317	1	N			gi|332021112	*Ac. echinatior*	5.00E-23
PPTAnchor1	Pp-346	1	N			gi|94468542	*Ae. aegypti*	3.00E-32
PPTAnchor2	Pp-348	1	N			gi|94468542	*Ae. aegypti*	3.00E-32
PPT unknown protein	Pp-571	1	N			gi|94468962	*Ae. aegypti*	4.00E-62
PPTGM23156	Pp-615	1	N			gi|195353883	*D. sechellia*	4.00E-65
PPTGJ12745	Pp-283	1	N			gi|195374720	*D. virilis*	7.00E-68
PPTGJ22064	Pp-579	1	N			gi|195383122	*D. virilis*	1.00E-115
PPTGE14742	Pp-552	1	N			gi|195471250	*D. yakuba*	3.00E-93
PPTGK19986	Pp-293	1	Y	21.263	4.57	gi|195432166	*D. willistoni*	2.00E-22
**TIMP-3 like protein**								
PPTTIMP	Pp-588	1	Y	26.65	9.3	gi|76446619	*Ae. aegypti*	3.00E-50

### Selection of Potential Markers of *P. papatasi* Exposure

Marzouki et al. [7] tested a number of sera from individuals living in an endemic area where *P. papatasi* is prevalent. These sera recognized a number of *P. papatasi* salivary proteins including proteins of 12, 15, 21, 28, 30, 36, 42, and 44 kDa. We have selected the following secreted proteins from the current SG transcriptome as potential markers of *P. papatasi* exposure based on the molecular weight of the proteins detected by individuals exposed to this sand fly, the predicted molecular weight of the transcripts identified in the current work, and whether the selected molecules have high degree of homology (if data are available) from strains of different geographic regions:

### Candidates for the 12- and 15-kDa Proteins

#### Small members of the OBP family–PPTSP12, PPTSP14, PPTSP14.2, PPTSP14.5, and PPTSP15

**Figure 11:**
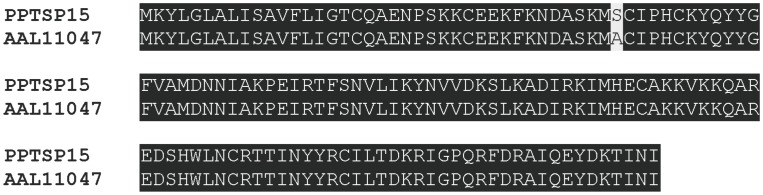
Sequence alignment of PPTSP15 with the PPSP15 from *Phlebotomus papatasi* Israeli strain (PpAAL11047). Black shading represents identical amino acids.

Marzouki et al. [7] showed that a protein with an approximate mw of 12 kDa induced the production of IgG antibodies in humans naturally exposed to *P. papatasi* bites. The isotypes of the induced antibodies (IgG1 and IgG2) were different from those induced by other immunogenic salivary proteins. The 12-kDa protein was not targeted by IgG4 antibodies, suggesting that the immune response induced by this protein was not polarized toward a Th2 phenotype [7]. If this 12-kDa protein is PPTSP12, this could represent a potential vaccine candidate or a marker of vector exposure. Another protein with an apparent mw of 15 kDa was shown to be immunogenic in humans [7]. This protein was recognized by IgG_1_, IgG_2_, and IgG_4_ antisaliva antibodies [7]. Further tests, including recombinant expression of this protein, are required to verify whether the PpSp15-like protein is the immunogenic protein in humans exposed to *P. papatasi* [7]. PPTSP15 is highly conserved and is almost identical to the PPSP15 from *P. papatasi* Israeli strain (Figure 11), suggesting that it may work as a marker for exposure in different areas where *P. papatasi* is prevalent. These data also suggest that–because of the predicted molecular weight of these small OBPs–any of these five proteins could be the immunogenic protein identified by western blot for the 12- or 15-kDa protein recognized by individuals living in *P. papatasi*-prevalent areas [7]. Because of the proximity of their molecular weight, it may be possible to identify these immunogenic antigens only by testing recombinant expression of each member of these families of proteins or by an immunoblot of a 2-D gel.

#### PPTSP16 family of proteins

This protein not previously reported in *P. papatasi* saliva has an mw very close to an immunogenic salivary protein of approximately15 kDa that was recognized by 90% of human sera exposed to *P. papatasi* [24].

### Candidate for the 21-kDa Protein

#### TIMP-3-like protein

We hypothesize this protein may not be a secreted protein from the SGs; however, it is interesting to note that the predicted mw of this protein is similar to the 21-kDa immunoreactive protein described by Marzouki et al. [7]. Further analyses are needed to address this issue.

### Candidates for the 28- and 30-kDa Proteins

#### D7 salivary protein PPTSP28 and PPTSP30

**Figure 12:**
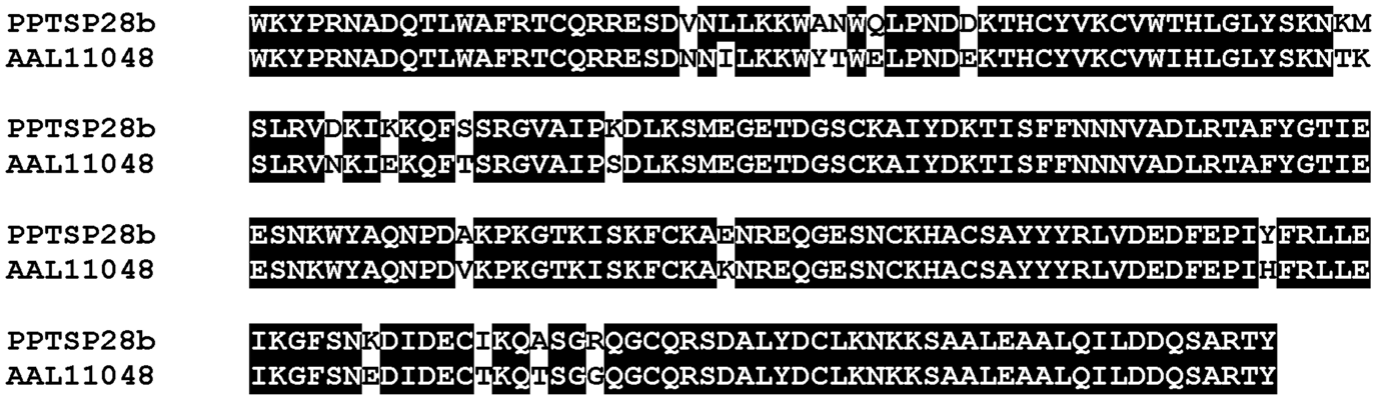
Alignment of PPTSP28b with the 28-kDa D7 protein from *Phlebotomus papatasi* Israeli strain (AAL11048). Black background shading represents identical amino acids.

Antibodies (IgG) specifically recognizing sand fly D7 proteins were found in dogs naturally exposed to Lu. longipalpis sand flies [38], [39]. Furthermore, it was recently shown that a salivary protein of approximately 30 kDa was an immunodominant salivary protein from *P. papatasi* in humans exposed to this sand fly [7]. It is possible that PPTSP28 and/or PPTSP30, which run at similar molecular weights, could be this immunodominant antigen in humans. We also tested the level of homology between D7 proteins from *P. papatasi* from different geographic regions (Tunisia and Israel). Sequence comparison between PPTSP30 and the D7 protein of 30 kDa from *P. papatasi* Israeli strain shows only one amino acid difference (Figure 2). Sequence comparison between the PPTSP28 and the D7 of 28 kDa from the Israeli strain shows more differences (Figure 12); however, these differences may not be significant enough (only 19 aa across the 235-aa molecule) to discard this protein as a potential marker of *P. papatasi* exposure in different regions. Due to the high transcript abundance of PPTSP28 compared with PPTSP30, PPTSP28 may be highly expressed in the saliva of *P. papatasi* and therefore a better candidate as a marker of vector exposure.

#### Antigen 5-related protein PPTSP29

**Figure 13:**
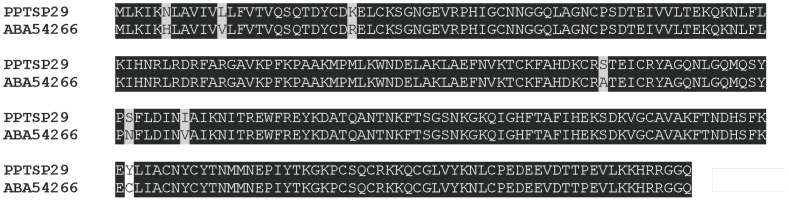
Sequence alignment of PPTSP29 with the Antigen-5 related protein from *Phlebotomus papatasi* Israeli strain (PpABA54266). Black shading representing identical amino acids.

The proximity of the mw (29 kDa) of PPTSP29 to that of the salivary protein with an apparent mw of 30 kDa that is recognized by sera of humans exposed to *P. papatasi* [7] makes PPTSP29 protein a good candidate as a marker for vector exposure. Furthermore, the level of identity between the PPTSP29 protein from the Tunisian strain and the Israeli strain is remarkably high (Figure 13), suggesting this protein could be used as a marker in different geographical areas where *P. papatasi* is prevalent.

#### PPTSP32 protein

Because of the proximity in mw of PPTSP32 to that of the 30-kDa protein recognized by people exposed to *P. papatasi*, PPTSP32 represents a good candidate for a marker of *P. papatasi* exposure. Furthermore, PPTSP32 shows high identity with the salivary PPSP32 from *P*. *papatasi* Israeli strain (data not shown).

### Candidates for the 36-kDa Proteins

#### PPTSP34 sand-fly anticoagulant

The predicted mw of *P. papatasi* anticoagulant (PPTSP34) is 34 kDa, a mw very close to that of the reported protein of similar mw (36 kDa) recognized by sera of individuals naturally exposed naturally to *P. papatasi* [7]. This family of proteins appears to be specific to sand flies.

#### PPTSP36/apyrase protein

The predicted molecular weight of *P. papatasi* apyrase (PPTSP36) is 36 kDa a molecular weight very close to the molecular weight recognized by sera of individuals naturally exposed naturally to *P. papatasi* [7]. Furthermore, PPTSP36 shows 98% identity with the salivary apyrase from *P. papatasi* Israeli strain (data not shown).

### Candidates for the 42- and 44 kDa Proteins

#### Yellow-related proteins PPTSP42 and PPTSP44

Sand-fly yellow proteins are proteins of approximately 42 and 44 kDa and were previously shown to be antigens recognized by sera of mice [40] and dogs experimentally bitten by sand flies [41] and humans naturally exposed to *Lu. longipalpis* bites [42]. Recently, Marzouki et al. described a protein with a relative mw of 44 kDa as one of the target molecules of the humoral immune response in humans [7]. Therefore, PPTSP42 and PPTSP44 are good candidates for markers of *P. papatasi* exposure.

#### PPTSP38.8 family of proteins

PPTPS38.8 is a protein of approximately 39 kDa and falls within the range of the 42-kDa protein that was shown to be antigenic from the saliva of *P. papatasi*. PPTSP38.8 is therefore a good candidate for the antigen that runs at 42 kDa.

### Potential *P. papatasi* Salivary Immunogens for Humans

Individuals naturally exposed to *P. papatasi* bites in Tunisia can mount a cellular immune response to the salivary proteins of this sand fly [6]. PBMCs isolated from these individuals produced T_H_1- and T_H_2-like responses after stimulation with *P. papatasi* SGH [6]. Because the resultant cellular immune response appears to be induced by T cells [6], it is not straightforward to predict which proteins may be responsible for inducing these immune responses. Furthermore, preliminary results suggest that the potential inducer(s) of cellular immune response in human PBMCs is in the fraction of salivary proteins with an mw of or above 30 kDa (data not shown). Although this probably narrows the number of candidate molecules, this still represents a mixture of a relatively good number of proteins. The results of this transcriptomic analysis generated a catalog of molecules that can then be tested in human PBMCs to identify these immunogens. We hypothesize that any of the transcripts coding for secreted proteins above 30 kDa are potential immunogens for T cells. These candidates can be tested either as recombinant proteins or as DNA plasmids in human PBMCs. A similar approach was successfully used in the selection of candidate molecules that induce a cellular immune response from the saliva of *Lu. longipalpis* in hamsters [43] and dogs [44], and this was because of the data available from the salivary transcriptome of *Lu. longipalpis* [29].

### *P. papatasi* Salivary Proteins as Potential Markers of Vector Exposure and Immunogens in Different Geographic Areas

In the present work, when we compared the protein sequences of the secreted salivary proteins of *P. papatasi* from different strains (Tunisian and Israeli), the level of amino acid identity was very high between these proteins (Figures 2, 11, 12, 13, and data not shown). This suggests that these proteins–if they are immunogenic (as makers or as inducers of T cell responses)–can be used in different geographic areas where *P. papatasi* is present. This level of identity was also demonstrated when comparing SG transcripts of *P. duboscqi* from Mali and Kenya [12]. This may suggest that intraspecies sand-fly salivary proteins are not highly divergent regardless of geographic distance. *P. papatasi* was shown to produce a DTH response in animals and humans [45]. Under laboratory conditions, this immune response helped the sand fly to probe and feed faster in DTH sites in human skin as compared with normal sites [45]. Therefore, maintaining the homology of certain salivary proteins, on an evolutionary scale, would be advantageous for sand-fly species by allowing the sand fly to probe and feed faster thereby increasing the chances of survival in nature.

### Conclusions

Overall, this transcriptomic analysis has increased our knowledge of the repertoire of proteins present in the saliva of the sand fly *P. papatasi*. We identified a number of salivary proteins never before described in *P. papatasi* including the homolog of Lufaxin, a novel factor Xa inhibitor from the saliva of *Lu. longipalpis* that appears to be exclusive to sand flies and the Aegyptin-like protein (PPTSP38.8) that may interact to collagen. We expanded the number of the small members of ODPs and presented evidence of their relatedness to sand-fly D7 proteins. A number of secreted proteins are being pursued as potential markers of *P. papatasi* exposure, and many of these proteins could be of use in different geographic areas. Expression of recombinant protein will be necessary to validate the proposed molecules as functional markers of *P. papatasi* exposure. This work represents a comprehensive data set that is essential for future studies related to developing epidemiologic tools to measure vector exposure and vector-based vaccines and discovering novel pharmacoactive proteins.

## Materials and Methods

### Sand-Fly SG Dissection

The colony of *P. papatasi* originated from El Felta–an endemic focus of zoonotic cutaneous leishmaniasis located in the governorate of Sidi Bouzid in Central Tunisia [9]–was reared in the insectary of Institut Pasteur de Tunis under standard conditions. Before mRNA extraction, SGs of 1- to 2-day-old females were dissected in saline buffer and stored in RNAlater (Qiagen, Santa Clara, California, USA) at 4°C.

### Construction of SG cDNA Library

SG mRNA was isolated from 50 pairs of SGs using Micro-FastTrack™ mRNA isolation kit (Invitrogen, San Diego, California, USA). PCR-based cDNA library was performed following the manufacturer’s instructions for the SMART™ cDNA library construction kit (BD Clontech, Palo Alto, California, USA) with some modifications as previously described [29]. The cDNA library was fractionated into three sets of cDNAs containing large, medium, and small fragments and visualized on an agarose gel.

Gigapack® III gold packaging extract (Stratagene, La Jolla, California, USA) was used for packaging phage particles. The libraries (large, medium, and small) were plated by infecting log-phase XL-1 blue *Escherichia coli* (Clontech). Several plaques from each plate were selected, and a PCR with selected vector-specific primers flanking the inserted cDNA was performed [12]. The presence of recombinants was checked by visualization of the PCR products on 1.1% agarose gel with Syber safe (Roche Diagnostics, Mannheim, Germany).

### Sequencing of Selected cDNA Clones

Plaques were randomly selected from the plated libraries and transferred to a 96 well-polypropylene plate containing 30 µl of water per well. The PCR reaction amplified randomly selected cDNAs using FastStart PCR Master Mix (Roche), 3 µl of the phage sample as a template, and the specific vector primers PT_2_F_1_ (5′-AAG TAC TCT AGC AAT TGT GAG C-3′), which is positioned upstream from the cDNA of interest (5′ end), and PT_2_R_1_ (5′- CTC TTC GCT ATT ACG CCA GCT-3′), which is positioned downstream from the cDNA of interest (3′ end). Amplification conditions were as follows: 1 hold of 75°C for 3 min, 1 hold of 94°C for 4 min, and 30 cycles of 94°C for 1 min, 49°C for 1 min, and 72°C for 2 min. The final elongation step lasted for 7 min at 72°C. Reaction products were cleaned using ExcelaPure 96-well UF PCR purification plates (EdgeBiosystems, Gaithersburg, Maryland, USA) and used as templates for cycle-sequencing reaction. Cycle sequencing reactions were performed at the Research Technology Branch at the Rocky Mountain Labs, NIAID.

### Bioinformatics

Bioinformatic analysis was performed as previously described [11]. Briefly, raw sequence data files were analyzed using a customized program [35]. DNA sequences with Phred quality scores lower than 20 were discarded, as were primer and vector sequences. The “cleaned” sequences were grouped into clusters using a customized program based on identity (95% identity) and aligned into contiguous sequences (contigs) using the CAP3 program [46]. The generated contigs were then analyzed by blastx, blastn, or rpsblast programs and compared with the non-redundant (NR) protein database of the NCBI, the gene ontology (GO) fasta subset, and the conserved domains database (CDD) of NCBI, which contains KOG, Pfam, and SMART databases. They were also compared with mitochondrial and rRNA nucleotide sequences available from NCBI. The three potential translations of each data set were submitted to the SignalP server to detect signal peptides. All the analyzed sequences were combined in an Excel spreadsheet and manually verified and annotated. N- and O-glycosylation site prediction was performed for selected sequences using NetNGlyc 1.0 and NetOGlyc 3.1 software.

### Sequence Alignment

Multiple sequence alignment of putative peptides was performed using Clustal Omega [33] and pairwise peptide alignments were accomplished using ClustalX2 [34]. Alignment outputs were converted to rich text files for figure annotation using BioEdit [28].

### Phylogenetic Analysis

ProtTest1.3 was used to evaluate the most appropriate model for protein evolution for Maximum Likelihood calculations [42] using the Phyml program [40] and PAL library [41]. The evolutionary history was inferred by using the Maximum Likelihood method based on the Jones-Taylor-Thorton (JTT) [47] or Whelan and Goldman (WAG) models [48], as suggested by ProtTest1.3. Evolutionary analyses were conducted with MEGA 5 [37]. The tree with the highest log likelihood is shown, and the percentage of trees in which the associated taxa clustered together is shown next to the branches. he tree is drawn to scale, with branch lengths measured in the number of substitutions per site. All positions containing gaps and missing data were eliminated.

## Supporting Information

Table S1 **Families of secreted proteins from salivary glands of Phlebotomus papatasi Tunisian strain.** (Only full-length sequences are shown in this table. Transcripts not described before are highlighted in grey.)
(DOC)

Table S2 **Non-salivary gland proteins. Potentially midgut or other organs proteins.**
(DOC)
