# Supplementary material for: Updating the Salivary Gland Transcriptome of Phlebotomus papatasi (Tunisian Strain): The Search for Sand Fly-Secreted Immunogenic Proteins for Humans
Source: PLoS One. 2012 Nov 6;7(11):e47347. doi: 10.1371/journal.pone.0047347 (PMC3491003; doi:10.1371/journal.pone.0047347)
Supplement: Table S1 — Families of secreted proteins from salivary glands of Phlebotomus papatasi Tunisian strain. (Only full-length sequences are shown in this table. Transcripts not described before are highlighted in grey.) (DOC) [file pone.0047347.s001.doc]

**Table S1: Families of secreted proteins from salivary glands of *Phlebotomus papatasi* Tunisian strain**

(Only full-length sequences are shown in this table. Transcripts not described before are highlighted in grey)

|  | | | | | | **Putative mature protein** | | | | **Best match to NR database** | | | |
| --- | --- | --- | --- | --- | --- | --- | --- | --- | --- | --- | --- | --- | --- |
| **Sequence name** | **Accession number** | **Contig**  **Number** | **Seq**  **per contig** | | **Transcript length** | **SigP** | **MW** | **pI** | **Protein**  **Length (AA)** | **Best Match** | **Species of best match** | | **E-value** |
| **OBP SP12-like family of proteins** | | | | | | | | | | | | | |
| PPTSP12 | JQ988874 | Pp-38 | 24 | | 545 | Y | 13.853 | 9.37 | 140 | [gi|15963505](http://www.ncbi.nlm.nih.gov/protein/15963505) | | *P. papatasi* | [2E-075](http://www.ncbi.nlm.nih.gov/sutils/blink.cgi?pid=15963505) |
| PPTSP12 | JQ988874 | Pp-39 | 8 | | 545 | Y | 13.853 | 9.37 | 140 | [gi|15963505](http://www.ncbi.nlm.nih.gov/protein/15963505) | | *P. papatasi* | [2E-075](http://www.ncbi.nlm.nih.gov/sutils/blink.cgi?pid=15963505) |
| PPTSP12 | JQ988874 | Pp-40 | 5 | | 534 | Y | 13.811 | 9.30 | 140 | [gi|15963505](http://www.ncbi.nlm.nih.gov/protein/15963505) | | *P. papatasi* | [3E-073](http://www.ncbi.nlm.nih.gov/sutils/blink.cgi?pid=15963505) |
| PPTSP12 | JQ988874 | Pp-41 | 3 | | 550 | Y | 13.827 | 9.39 | 140 | [gi|15963505](http://www.ncbi.nlm.nih.gov/protein/15963505) | | *P. papatasi* | [9E-075](http://www.ncbi.nlm.nih.gov/sutils/blink.cgi?pid=15963505) |
| **OBP SP14.2 family of proteins** | | | | | | | | | | | | | |
| PPTSP14.2a | JQ988876 | [Pp-90](../../../../C:%5CUsers%5Cmaha%5CDesktop%5Clinks%5Cpep%5CPp-90-pep.txt) | 7 | | 517 | Y | 14.185 | 7.76 | 141 | [gi|112497698](http://www.ncbi.nlm.nih.gov/protein/112497698) | | *P. duboscqi* | [7E-059](http://www.ncbi.nlm.nih.gov/sutils/blink.cgi?pid=112497698) |
| PPTSP14.2a | JQ988876 | [Pp-92](../../../../C:%5CUsers%5Cmaha%5CDesktop%5Clinks%5Cpep%5CPp-92-pep.txt) | 2 | | 548 | Y | 14.110 | 7.13 | 141 | [gi|112497698](http://www.ncbi.nlm.nih.gov/protein/112497698) | | *P. duboscqi* | [1E-052](http://www.ncbi.nlm.nih.gov/sutils/blink.cgi?pid=112497698) |
| PPTSP14.2b | JQ988877 | [Pp-97](../../../../C:%5CUsers%5Cmaha%5CDesktop%5Clinks%5Cpep%5CPp-97-pep.txt) | 5 | | 534 | Y | 14.172 | 7.72 | 141 | [gi|112496839](http://www.ncbi.nlm.nih.gov/protein/112496839) | | *P. duboscqi* | [2E-066](http://www.ncbi.nlm.nih.gov/sutils/blink.cgi?pid=112496839) |
| PPTSP14.2a | JQ988876 | [Pp-91](../../../../C:%5CUsers%5Cmaha%5CDesktop%5Clinks%5Cpep%5CPp-91-pep.txt) | 3 | | 553 | Y | 14.159 | 6.48 | 141 | [gi|112497698](http://www.ncbi.nlm.nih.gov/protein/112497698) | | *P. duboscqi* | [8E-060](http://www.ncbi.nlm.nih.gov/sutils/blink.cgi?pid=112497698) |
| **OBP SP14.5 like family of proteins** | | | | | | | | | | | | | |
| PPTSP14.5 | JQ988878 | Pp-30 | 13 | | 536 | Y | 14.542 | 9.39 | 142 | [gi|112497496](http://www.ncbi.nlm.nih.gov/protein/112497496) | | *P. duboscqi* | [2E-077](http://www.ncbi.nlm.nih.gov/sutils/blink.cgi?pid=112497496) |
| PPTSP14.5 | JQ988878 | Pp-29 | 7 | | 534 | Y | 14.511 | 9.32 | 142 | [gi|112497496](http://www.ncbi.nlm.nih.gov/protein/112497496) | | *P. duboscqi* | [2E-076](http://www.ncbi.nlm.nih.gov/sutils/blink.cgi?pid=112497496) |
| **OBP SP15 like family of proteins** | | | | | | | | | | | | | |
| PPTSP15 | JQ988879 | Pp-28 | 31 | 535 | | Y | 14.502 | 9.39 | 142 | [gi|15963509](http://www.ncbi.nlm.nih.gov/protein/15963509) | | *P. papatasi* | [2E-078](http://www.ncbi.nlm.nih.gov/sutils/blink.cgi?pid=15963509) |
| **OBP SP14 like family of proteins** | | | | | | | | | | | | | |
| PPTSP14 | JQ988880 | Pp-17 | 18 | 515 | | Y | 14.736 | 8.85 | 142 | [gi|15963507](http://www.ncbi.nlm.nih.gov/protein/15963507) | | *P. papatasi* | [8E-079](http://www.ncbi.nlm.nih.gov/sutils/blink.cgi?pid=15963507) |
| PPTSP14 | JQ988880 | Pp-16 | 11 | 731 | | Y | 14.806 | 8.87 | 142 | [gi|15963507](http://www.ncbi.nlm.nih.gov/protein/15963507) | | *P. papatasi* | [9E-080](http://www.ncbi.nlm.nih.gov/sutils/blink.cgi?pid=15963507) |
| PPTSP14 | JQ988880 | Pp-14 | 9 | 522 | | Y | 14.764 | 8.86 | 142 | [gi|15963507](http://www.ncbi.nlm.nih.gov/protein/15963507) | | *P. papatasi* | [1E-079](http://www.ncbi.nlm.nih.gov/sutils/blink.cgi?pid=15963507) |
| PPTSP14 | JQ988880 | Pp-22 | 9 | 513 | | Y | 14.794 | 8.86 | 142 | [gi|15963507](http://www.ncbi.nlm.nih.gov/protein/15963507) | | *P. papatasi* | [1E-078](http://www.ncbi.nlm.nih.gov/sutils/blink.cgi?pid=15963507) |
| PPTSP14 | JQ988880 | Pp-15 | 7 | 514 | | Y | 14.736 | 8.85 | 142 | [gi|15963507](http://www.ncbi.nlm.nih.gov/protein/15963507) | | *P. papatasi* | [8E-079](http://www.ncbi.nlm.nih.gov/sutils/blink.cgi?pid=15963507) |
| PPTSP14 | JQ988880 | Pp-13 | 3 | 514 | | Y | 14.776 | 8.86 | 142 | [gi|15963507](http://www.ncbi.nlm.nih.gov/protein/15963507) | | *P. papatasi* | [6E-079](http://www.ncbi.nlm.nih.gov/sutils/blink.cgi?pid=15963507) |
| PPTSP14 | JQ988880 | Pp-18 | 3 | 507 | | Y | 14.722 | 8.85 | 142 | [gi|15963507](http://www.ncbi.nlm.nih.gov/protein/15963507) | | *P. papatasi* | [6E-079](http://www.ncbi.nlm.nih.gov/sutils/blink.cgi?pid=15963507) |
| PPTSP14 | JQ988880 | Pp-19 | 3 | 514 | | Y | 14.754 | 8.86 | 142 | [gi|15963507](http://www.ncbi.nlm.nih.gov/protein/15963507) | | *P. papatasi* | [1E-078](http://www.ncbi.nlm.nih.gov/sutils/blink.cgi?pid=15963507) |
| PPTSP14 | JQ988880 | Pp20 | 3 | 553 | | Y | 14.794 | 8.86 | 142 | [gi|15963507](http://www.ncbi.nlm.nih.gov/protein/15963507) | | *P. papatasi* | [1E-078](http://www.ncbi.nlm.nih.gov/sutils/blink.cgi?pid=15963507) |
| PPTSP14 | JQ988880 | Pp-21 | 3 | 511 | | Y | 14.720 | 8.85 | 142 | [gi|15963507](http://www.ncbi.nlm.nih.gov/protein/15963507) | | *P. papatasi* | [3E-080](http://www.ncbi.nlm.nih.gov/sutils/blink.cgi?pid=15963507) |
| **OBP D7 SP28 like family of proteins** | | | | | | | | | | | | | |
| PPTSP28a | JQ988881 | Pp-3 | 82 | 922 | | Y | 27.365 | 9.04 | 254 | [gi|15963511](http://www.ncbi.nlm.nih.gov/protein/15963511) | | *P. papatasi* | [1E-136](http://www.ncbi.nlm.nih.gov/sutils/blink.cgi?pid=15963511) |
| PPTSP28b | JQ988882 | [Pp-1](../../../../C:%5CUsers%5Cmaha%5CDesktop%5Clinks%5Cpep%5CPp-1-pep.txt) | 21 | 900 | | Y | 27.258 | 8.96 | 254 | [gi|15963511](http://www.ncbi.nlm.nih.gov/protein/15963511) | | *P. papatasi* | [1E-137](http://www.ncbi.nlm.nih.gov/sutils/blink.cgi?pid=15963511) |
| PPTSP28a | JQ988881 | [Pp-4](../../../../C:%5CUsers%5Cmaha%5CDesktop%5Clinks%5Cpep%5CPp-4-pep.txt) | 9 | 896 | | Y | 27.304 | 8.70 | 254 | [gi|15963511](http://www.ncbi.nlm.nih.gov/protein/15963511) | | *P. papatasi* | [1E-139](http://www.ncbi.nlm.nih.gov/sutils/blink.cgi?pid=15963511) |
| PPTSP28c | JQ988883 | [Pp-5](../../../../C:%5CUsers%5Cmaha%5CDesktop%5Clinks%5Cpep%5CPp-5-pep.txt) | 8 | 915 | | Y | 27.315 | 8.43 | 254 | [gi|15963511](http://www.ncbi.nlm.nih.gov/protein/15963511) | | *P. papatasi* | [1E-141](http://www.ncbi.nlm.nih.gov/sutils/blink.cgi?pid=15963511) |
| PPTSP28b | JQ988882 | [Pp-2](../../../../C:%5CUsers%5Cmaha%5CDesktop%5Clinks%5Cpep%5CPp-2-pep.txt) | 3 | 941 | | Y | 27.229 | 9.09 | 254 | [gi|15963511](http://www.ncbi.nlm.nih.gov/protein/15963511) | | *P. papatasi* | [1E-138](http://www.ncbi.nlm.nih.gov/sutils/blink.cgi?pid=15963511) |
| PPTSP28b | JQ988882 | [Pp-6](../../../../C:%5CUsers%5Cmaha%5CDesktop%5Clinks%5Cpep%5CPp-6-pep.txt) | 3 | 905 | | Y | 27.309 | 8.57 | 254 | [gi|15963511](http://www.ncbi.nlm.nih.gov/protein/15963511) | | *P. papatasi* | [1E-141](http://www.ncbi.nlm.nih.gov/sutils/blink.cgi?pid=15963511) |
| PPTSP28b | JQ988882 | [Pp-7](../../../../C:%5CUsers%5Cmaha%5CDesktop%5Clinks%5Cpep%5CPp-7-pep.txt) | 3 | 900 | | Y | 27.336 | 8.43 | 254 | [gi|15963511](http://www.ncbi.nlm.nih.gov/protein/15963511) | | *P. papatasi* | [1E-137](http://www.ncbi.nlm.nih.gov/sutils/blink.cgi?pid=15963511) |
| **OBP D7 SP30 like family of proteins** | | | | | | | | | | | | | |
| PPTSP30 | JQ988884 | Pp-101 | 1 | 870 | | Y | 27.7 | 9.02 | 253 | gi|15963513 | | *P. papatasi* | 1E-146 |
| **Yellow PPSP42 like family of proteins** | | | | | | | | | | | | | |
| PPTSP42 | JQ988885 | Pp-51 | 9 | 1330 | | Y | 42.321 | 9.11 | 395 | [gi|15963517](http://www.ncbi.nlm.nih.gov/protein/15963517) | | *P. papatasi* | [0.0](http://www.ncbi.nlm.nih.gov/sutils/blink.cgi?pid=15963517) |
| PPTSP42 | JQ988885 | [Pp-52](../../../../C:%5CUsers%5Cmaha%5CDesktop%5Clinks%5Cpep%5CPp-52-pep.txt) | 3 | 1333 | | Y | 42.385 | 9.07 | 395 | [gi|15963517](http://www.ncbi.nlm.nih.gov/protein/15963517) | | *P. papatasi* | [0.0](http://www.ncbi.nlm.nih.gov/sutils/blink.cgi?pid=15963517) |
| **Yellow PPSP44 like family of proteins** | | | | | | | | | | | | | |
| PPTSP44 | JQ988886 | [Pp-35](../../../../C:%5CUsers%5Cmaha%5CDesktop%5Clinks%5Cpep%5CPp-35-pep.txt) | 32 | 1335 | | Y | 43.608 | 8.40 | 400 | [gi|15963519](http://www.ncbi.nlm.nih.gov/protein/15963519) | | *P. papatasi* | [0.0](http://www.ncbi.nlm.nih.gov/sutils/blink.cgi?pid=15963519) |
| PPTSP44 | JQ988886 | [Pp-34](../../../../C:%5CUsers%5Cmaha%5CDesktop%5Clinks%5Cpep%5CPp-34-pep.txt) | 17 | 1380 | | Y | 43.667 | 8.58 | 400 | [gi|15963519](http://www.ncbi.nlm.nih.gov/protein/15963519) | | *P. papatasi* | [0.0](http://www.ncbi.nlm.nih.gov/sutils/blink.cgi?pid=15963519) |
| **Antigen-5 PPSP29 family of proteins** | | | | | | | | | | | | | |
| PPTSP29 | JQ988887 | [Pp-64](../../../../C:%5CUsers%5Cmaha%5CDesktop%5Clinks%5Cpep%5CPp-64-pep.txt) | 9 | 1094 | | Y | 28.844 | 9.10 | 272 | [gi|76589378](http://www.ncbi.nlm.nih.gov/protein/76589378) | | *P. papatasi* | [1E-158](http://www.ncbi.nlm.nih.gov/sutils/blink.cgi?pid=76589378) |
| PPTSP29 | JQ988887 | [Pp-67](../../../../C:%5CUsers%5Cmaha%5CDesktop%5Clinks%5Cpep%5CPp-67-pep.txt) | 7 | 1009 | | Y | 28.673 | 9.04 | 272 | [gi|76589378](http://www.ncbi.nlm.nih.gov/protein/76589378) | | *P. papatasi* | [1E-157](http://www.ncbi.nlm.nih.gov/sutils/blink.cgi?pid=76589378) |
| PPTSP29 | JQ988887 | [Pp-68](../../../../C:%5CUsers%5Cmaha%5CDesktop%5Clinks%5Cpep%5CPp-68-pep.txt) | 6 | 1023 | | Y | 28.93 | 9.04 | 272 | [gi|76589378](http://www.ncbi.nlm.nih.gov/protein/76589378) | | *P. papatasi* | [1E-158](http://www.ncbi.nlm.nih.gov/sutils/blink.cgi?pid=76589378) |
| PPTSP29 | JQ988887 | [Pp-66](../../../../C:%5CUsers%5Cmaha%5CDesktop%5Clinks%5Cpep%5CPp-66-pep.txt) | 3 | 1001 | | Y | 28.884 | 9.16 | 272 | [gi|76589378](http://www.ncbi.nlm.nih.gov/protein/76589378) | | *P. papatasi* | [1E-158](http://www.ncbi.nlm.nih.gov/sutils/blink.cgi?pid=76589378) |
| **Silk-related SP32 like family of proteins** | | | | | | | | | | | | | |
| PPTSP32 | JQ988888 | [Pp-63](../../../../C:%5CUsers%5Cmaha%5CDesktop%5Clinks%5Cpep%5CPp-63-pep.txt) | 25 | 886 | | Y | 24.465 | 8.95 | 246 | [gi|15963515](http://www.ncbi.nlm.nih.gov/protein/15963515) | | *P. papatasi* | [1E-137](http://www.ncbi.nlm.nih.gov/sutils/blink.cgi?pid=15963515) |
| PPTSP32 | JQ988888 | [Pp-62](../../../../C:%5CUsers%5Cmaha%5CDesktop%5Clinks%5Cpep%5CPp-62-pep.txt) | 11 | 883 | | Y | 24.493 | 9.30 | 246 | [gi|15963515](http://www.ncbi.nlm.nih.gov/protein/15963515) | | *P. papatasi* | [1E-134](http://www.ncbi.nlm.nih.gov/sutils/blink.cgi?pid=15963515) |
| PPTSP32 | JQ988888 | [Pp-61](../../../../C:%5CUsers%5Cmaha%5CDesktop%5Clinks%5Cpep%5CPp-61-pep.txt) | 2 | 891 | | Y | 24.519 | 8.95 | 246 | [gi|15963515](http://www.ncbi.nlm.nih.gov/protein/15963515) | | *P. papatasi* | [1E-135](http://www.ncbi.nlm.nih.gov/sutils/blink.cgi?pid=15963515) |
| **SP34 protein. Family of sand fly anticoagulant proteins** | | | | | | | | | | | | | |
| PPTSP34 | JQ988889 | [Pp-73](../../../../C:%5CUsers%5Cmaha%5CDesktop%5Clinks%5Cpep%5CPp-73-pep.txt) | 8 | 1109 | | Y | 34.07 | 9.21 | 313 | [gi|112496879](http://www.ncbi.nlm.nih.gov/protein/112496879) | | *P. duboscqi* | [1E-143](http://www.ncbi.nlm.nih.gov/sutils/blink.cgi?pid=112496879) |
| **SP56.6 like family of proteins** | | | | | | | | | | | | | |
| PPTSP56.6 | JQ988890 | [Pp-104](../../../../C:%5CUsers%5Cmaha%5CDesktop%5Clinks%5Cpep%5CPp-104-pep.txt) | 2 | 1537 | | Y | 50.12 | 4.57 | 471 | [gi|299829444](http://www.ncbi.nlm.nih.gov/protein/299829444) | | *P. sergenti* | [1E-172](http://www.ncbi.nlm.nih.gov/sutils/blink.cgi?pid=299829444) |
| **Alpha amylase family of proteins** | | | | | | | | | | | | | |
| PPTAMY | JQ988891 | [Pp-55](../../../../C:%5CUsers%5Cmaha%5CDesktop%5Clinks%5Cpep%5CPp-55-pep.txt) | 11 | 1727 | | Y | 54.02 | 6.50 | 497 | [gi|4887104](http://www.ncbi.nlm.nih.gov/protein/4887104) | | *L.longipalpis* | [0.0](http://www.ncbi.nlm.nih.gov/sutils/blink.cgi?pid=4887104) |
| **Apyrase SP36 like family of proteins** | | | | | | | | | | | | | |
| PPTSP36 | JQ988892 | [Pp-76](../../../../C:%5CUsers%5Cmaha%5CDesktop%5Clinks%5Cpep%5CPp-76-pep.txt) | 8 | 1121 | | Y | 35.90 | 9.03 | 336 | [gi|10443907](http://www.ncbi.nlm.nih.gov/protein/10443907) | | *P. papatasi* | [0.0](http://www.ncbi.nlm.nih.gov/sutils/blink.cgi?pid=10443907) |
| PPTSP36 | JQ988892 | [Pp-75](../../../../C:%5CUsers%5Cmaha%5CDesktop%5Clinks%5Cpep%5CPp-75-pep.txt) | 6 | 1105 | | Y | 36.00 | 9.03 | 336 | [gi|10443907](http://www.ncbi.nlm.nih.gov/protein/10443907) | | *P. papatasi* | [0.0](http://www.ncbi.nlm.nih.gov/sutils/blink.cgi?pid=10443907) |
| PPTSP36 | JQ988892 | [Pp-77](../../../../C:%5CUsers%5Cmaha%5CDesktop%5Clinks%5Cpep%5CPp-77-pep.txt) | 6 | 1106 | | Y | 35.91 | 9.03 | 336 | [gi|10443907](http://www.ncbi.nlm.nih.gov/protein/10443907) | | *P. papatasi* | [0.0](http://www.ncbi.nlm.nih.gov/sutils/blink.cgi?pid=10443907) |
| **SP16 like family of proteins** | | | | | | | | | | | | | |
| PPTSP14.3 | JQ988893 | [Pp-413](../../../../C:%5CUsers%5Cmaha%5CDesktop%5Clinks%5Cpep%5CPp-413-pep.txt) | 1 | 760 | | Y | 14.06 | 4.82 | 159 | [gi|299829434](http://www.ncbi.nlm.nih.gov/protein/299829434) | | *P. sergenti* | [8E-068](http://www.ncbi.nlm.nih.gov/sutils/blink.cgi?pid=299829434) |
| **SP2.5 kDa like family of proteins** | | | | | | | | | | | | | |
| PPTSP2.5 | JQ988875 | Pp-147 | 1 | 1008 | | Y | 3.1 | 10.6 | 49 | gi|112497575 | | *P. duboscqi* | 3E-050 |
| **SP38.8 kDa like family of proteins** | | | | | | | | | | | | | |
| PPTSP38.8 |  | Pp-219 | 1 | 1022 | | Y | 36.80 | 4.37 | 341 | gi|299829376 | | *P. tobbi* | 2E-050 |
