# Supplementary material for: Updating the Salivary Gland Transcriptome of Phlebotomus papatasi (Tunisian Strain): The Search for Sand Fly-Secreted Immunogenic Proteins for Humans
Source: PLoS One. 2012 Nov 6;7(11):e47347. doi: 10.1371/journal.pone.0047347 (PMC3491003; doi:10.1371/journal.pone.0047347)
Supplement: Table S2 — Non-salivary gland proteins. Potentially midgut or other organs proteins. (DOC) [file pone.0047347.s002.doc]

**TableS2: Non-salivary gland proteins. Potentially midgut or other organs proteins**

|  |  | |  |  | | | **Putative mature protein** | | | **Best match to NR database** | | |  |
| --- | --- | --- | --- | --- | --- | --- | --- | --- | --- | --- | --- | --- | --- |
| **Sequence name** | **Contig**  **Number** | **Number of**  **Sequences**  **per contig** | | | **SigP** | **MW** | | **pI** | **Best Match** | | **Species of best match** | **E-value** | |
| **Midgut proteins** | | | | | | | | | | | | |  |
| PPT11.6 | [Pp-202](../links/pep/Pp-202-pep.txt) | 1 | | | N | 11.900 | | 9.27 | [gi|157361609](http://www.ncbi.nlm.nih.gov/protein/157361609) | | *P. papatasi* | [3E-053](http://www.ncbi.nlm.nih.gov/sutils/blink.cgi?pid=157361609) | |
| PPT13.6 | [Pp-566](../links/pep/Pp-566-pep.txt) | 1 | | | Y | 11.598 | | 10 | [gi|157361609](http://www.ncbi.nlm.nih.gov/protein/157361609) | | *P. papatasi* | [2E-065](http://www.ncbi.nlm.nih.gov/sutils/blink.cgi?pid=157361565) | |
| PPTmicrovilli-like | [Pp-404](../links/pep/Pp-404-pep.txt) | 1 | | | Y | 23.707 | | 5.28 | [gi|157361605](http://www.ncbi.nlm.nih.gov/protein/157361605) | | *P. papatasi* | [1E-118](http://www.ncbi.nlm.nih.gov/sutils/blink.cgi?pid=157361605) | |
| PPTmicrovilli-like | [Pp-565](../links/pep/Pp-565-pep.txt) | 1 | | | Y | 23.779 | | 5.43 | [gi|157361605](http://www.ncbi.nlm.nih.gov/protein/157361605) | | *P. papatasi* | [1E-119](http://www.ncbi.nlm.nih.gov/sutils/blink.cgi?pid=157361605) | |
| **Extracellular matrix** | | | | | | | | | | | | |  |
| PPTGalectin | Pp-493 | 1 | | | N | 10.491 | | 8.66 | [gi|47121805](http://www.ncbi.nlm.nih.gov/protein/47121805) | | *P. papatasi* | [0.016](http://www.uniprot.org/uniprot/O54891) | |
| **Serine protease** | | | | | | | | | | | | |  |
| PPTtrypsin 1 | Pp-115 | 1 | | | Y | 28.442 | | 5.03 | [gi|32394738](http://www.ncbi.nlm.nih.gov/protein/32394738) | | *P. papatasi* | [1E-148](http://www.ncbi.nlm.nih.gov/sutils/blink.cgi?pid=32394738) | |
| **Peritrophin-like protein** | | | | | | | | | | | | |  |
| PPTperitrophin-like | Pp-102 | 1 | | | Y | 9.648 | | 4.4 | [gi|157361591](http://www.ncbi.nlm.nih.gov/protein/157361591) | | *P. papatasi* | [8E-047](http://www.ncbi.nlm.nih.gov/sutils/blink.cgi?pid=157361591) | |
| **Hypothetical protein** | | | | | | | | | | | | |  |
| PPT[AND_04019](../links/NR/Pp-614-NR.txt) | [Pp-614](../links/pep/Pp-614-pep.txt) | 1 | | | N |  | |  | [gi|312383031](http://www.ncbi.nlm.nih.gov/protein/312383031) | | *An. darlingi* | [2E-011](http://www.ncbi.nlm.nih.gov/sutils/blink.cgi?pid=312383031) | |
| PPTAaeL008425 | [Pp-478](../links/pep/Pp-478-pep.txt) | 1 | | | N | 15.322 | | 5.29 | [gi|157118844](http://www.ncbi.nlm.nih.gov/protein/157118844) | | *Ae. aegypti* | [1E-065](http://www.ncbi.nlm.nih.gov/sutils/blink.cgi?pid=157118844) | |
| [PPTAaeL012123](../links/NR/Pp-376-NR.txt) | [Pp-376](../links/pep/Pp-376-pep.txt) | 1 | | | Y | 18.615 | | 9.92 | [gi|157131504](http://www.ncbi.nlm.nih.gov/protein/157131504) | | *Ae. aegypti* | [2E-095](http://www.ncbi.nlm.nih.gov/sutils/blink.cgi?pid=157131504) | |
| [PPTAND_22328](../links/NR/Pp-223-NR.txt) | [Pp-223](../links/pep/Pp-223-pep.txt) | 1 | | | N |  | |  | [gi|312371260](http://www.ncbi.nlm.nih.gov/protein/312371260) | | *An. darlingi* | [5E-027](http://www.ncbi.nlm.nih.gov/sutils/blink.cgi?pid=312371260) | |
| **Conserved hypothetical protein** | | | | | | | | | | | | |  |
| PPTH1 | [Pp-524](../links/pep/Pp-524-pep.txt) | 1 | | | N |  | |  | [gi|170069526](http://www.ncbi.nlm.nih.gov/protein/170069526) | | *C. quinquefasciatus* | [1E-043](http://www.ncbi.nlm.nih.gov/sutils/blink.cgi?pid=170069526) | |
| PPTH2 | [Pp-195](../links/pep/Pp-195-pep.txt) | 1 | | | N |  | |  | [gi|170032716](http://www.ncbi.nlm.nih.gov/protein/170032716) | | *C. quinquefasciatus* | [3E-040](http://www.ncbi.nlm.nih.gov/sutils/blink.cgi?pid=170032716) | |
| **Other proteins** | | | | | | | | | | | | |  |
| PPT[AGAP012418-PA](../links/NR/Pp-426-NR.txt) | [Pp-426](../links/pep/Pp-426-pep.txt) | 1 | | | N |  | |  | [gi|58393517](http://www.ncbi.nlm.nih.gov/protein/58393517) | | *An.gambiae* | [1E-014](http://www.ncbi.nlm.nih.gov/sutils/blink.cgi?pid=58393517) | |
| PPT[FAM8A1](../links/NR/Pp-608-NR.txt) | [Pp-608](../links/pep/Pp-608-pep.txt) | 1 | | | N |  | |  | [gi|170036645](http://www.ncbi.nlm.nih.gov/protein/170036645) | | *C. quinquefasciatus* | [6E-051](http://www.ncbi.nlm.nih.gov/sutils/blink.cgi?pid=170036645) | |
| PPT[25](../links/NR/Pp-317-NR.txt) | [Pp-317](../links/pep/Pp-317-pep.txt) | 1 | | | N |  | |  | [gi|332021112](http://www.ncbi.nlm.nih.gov/protein/332021112) | | *Ac. echinatior* | [5E-023](http://www.ncbi.nlm.nih.gov/sutils/blink.cgi?pid=332021112) | |
| PPTAnchor1 | [Pp-346](../links/pep/Pp-346-pep.txt) | 1 | | | N |  | |  | [gi|94468542](http://www.ncbi.nlm.nih.gov/protein/94468542) | | Ae. aegypti | [3E-032](http://www.ncbi.nlm.nih.gov/sutils/blink.cgi?pid=94468542) | |
| PPTAnchor2 | [Pp-348](../links/pep/Pp-348-pep.txt) | 1 | | | N |  | |  | [gi|94468542](http://www.ncbi.nlm.nih.gov/protein/94468542) | | *Ae. aegypti* | [3E-032](http://www.ncbi.nlm.nih.gov/sutils/blink.cgi?pid=94468542) | |
| PPT[unknown protein](../links/NR/Pp-571-NR.txt) | [Pp-571](../links/pep/Pp-571-pep.txt) | 1 | | | N |  | |  | [gi|94468962](http://www.ncbi.nlm.nih.gov/protein/94468962) | | *Ae. aegypti* | [4E-062](http://www.ncbi.nlm.nih.gov/sutils/blink.cgi?pid=94468962) | |
| PPT[GM23156](../links/NR/Pp-615-NR.txt) | [Pp-615](../links/pep/Pp-615-pep.txt) | 1 | | | N |  | |  | [gi|195353883](http://www.ncbi.nlm.nih.gov/protein/195353883) | | *D. sechellia* | [4E-065](http://www.ncbi.nlm.nih.gov/sutils/blink.cgi?pid=195353883) | |
| PPT[GJ12745](../links/NR/Pp-283-NR.txt) | [Pp-283](../links/pep/Pp-283-pep.txt) | 1 | | | N |  | |  | [gi|195374720](http://www.ncbi.nlm.nih.gov/protein/195374720) | | *D. virilis* | [7E-068](http://www.ncbi.nlm.nih.gov/sutils/blink.cgi?pid=195374720) | |
| PPT[GJ22064](../links/NR/Pp-579-NR.txt) | [Pp-579](../links/pep/Pp-579-pep.txt) | 1 | | | N |  | |  | [gi|195383122](http://www.ncbi.nlm.nih.gov/protein/195383122) | | *D. virilis* | [1E-115](http://www.ncbi.nlm.nih.gov/sutils/blink.cgi?pid=195383122) | |
| PPT[GE14742](../links/NR/Pp-552-NR.txt) | [Pp-552](../links/pep/Pp-552-pep.txt) | 1 | | | N |  | |  | [gi|195471250](http://www.ncbi.nlm.nih.gov/protein/195471250) | | *D. yakuba* | [3E-093](http://www.ncbi.nlm.nih.gov/sutils/blink.cgi?pid=195471250) | |
| PPT[GK19986](../links/NR/Pp-293-NR.txt) | [Pp-293](../links/pep/Pp-293-pep.txt) | 1 | | | Y | 21.263 | | 4.57 | [gi|195432166](http://www.ncbi.nlm.nih.gov/protein/195432166) | | *D. willistoni* | [2E-022](http://www.ncbi.nlm.nih.gov/sutils/blink.cgi?pid=195432166) | |
| **TIMP-3 like protein** | | | | | | | | | | | | |  |
| PPTTIMP | Pp-588 | 1 | | | Y | 26.65 | | 9.3 | gi|76446619 | | *Ae. aegypti* | 3E-050 | |
